# Seeing inferences: brain dynamics and oculomotor signatures of non-verbal deduction

**DOI:** 10.1038/s41598-023-29307-3

**Published:** 2023-02-09

**Authors:** Ana Martín-Salguero, Carlo Reverberi, Aldo Solari, Luca Filippin, Christophe Pallier, Luca L. Bonatti

**Affiliations:** 1 Center for Brain and Cognition, Department of Information and Communications Technologies, Universitat Pompeu Fabra, Ramon Trias Fargas, 25-27, 08005 Barcelona, Spain; 2 Institut Jean Nicod, Département d’Études Cognitives, Ecole Normale Supérieure, EHESS, CNRS, PSL University, 29 Rue d’Ulm, 75005 Paris, France; 3 NeuroMI-Milan Center for Neuroscience and Departments of Psychology, University of Milano–Bicocca, 20126 Milan, Italy; 4 Department of Psychology, University of Milano-Bicocca, 20126 Milan, Italy; 5 Milan Center for Neuroscience, University of Milano-Bicocca, 20126 Milan, Italy; 6 Department of Economics, Management and Statistics, University of Milano–Bicocca, 20126 Milan, Italy; 7 Cognitive Neuroimaging Unit U992, Institut National de la Santé et de la Recherche Médicale, Commissariat à l’Énergie Atomique et aux Énergies Alternatives, Direction de la Recherche Fondamentale/Institut Joliot, Centre National de la Recherche Scientifique ERL9003, NeuroSpin Center, Université Paris-Saclay, 91191 Gif-Sur-Yvette, France; 8 ICREA, Pg. Lluís Companys 23, 08010 Barcelona, Spain

**Keywords:** Problem solving, Human behaviour, Decision

## Abstract

We often express our thoughts through words, but thinking goes well beyond language. Here we focus on an elementary but basic thinking process, disjunction elimination, elicited by elementary visual scenes deprived of linguistic content, describing its neural and oculomotor correlates. We track two main components of a nonverbal deductive process: the construction of a logical representation (A or B), and its simplification by deduction (not A, therefore B). We identify the network active in the two phases and show that in the latter, but not in the former, it overlaps with areas known to respond to verbal logical reasoning. Oculomotor markers consistently differentiate logical processing induced by the construction of a representation, its simplification by deductive inference, and its maintenance when inferences cannot be drawn. Our results reveal how integrative logical processes incorporate novel experience in the flow of thoughts induced by visual scenes.

## Introduction

Humans think. They make sense of their world and modify it by having ideas, inventing solutions to problems, learning from their failures, and generating novel ideas. All this would be impossible without,* inter alia*, the ability to carry out elementary deductions. I see someone at a distance who looks like my son; but I just dropped him off at school, so I continue on my way to the office. Reasonings of this kind, involving some process of deductive elimination, are ubiquitous in everyday life, often prompted by perception. They are no different from how scientists explore hypotheses or infants learn novel facts or words^1–3^. In this respect, learning processes, common sense, or scientific inquiry equally depend on elementary deductions. The centrality and ubiquity of spontaneous logical reasoning explain why psychologists and philosophers tried to understand it since the inception of their disciplines, with mixed success. The aim of this paper is to study the dynamics of such spontaneous logical processes when participants watch ordinary scenes not accompanied by linguistic descriptions. We know very little about these processes: deduction has been mostly studied using verbally presented problems, often given in written form, where some sentences are explicitly identified as premises and others as conclusions to be evaluated^4,5^. However, while we often dress our thinking with verbal clothes, we also reason when we organize our everyday's experience, solving the myriads of little puzzles that life constantly presents us. The few studies involving nonverbal reasoning are of little help to advance our understanding of the role of logic in everyday experience. Some of them focus on complex problems such as Raven matrices or variations of them^6–8^, in which logical processes occur inextricably mixed with other forms of reasoning, such as analogical or mathematical; as such, they are too coarse to gauge the specific role of elementary deduction. Other reasoning studies focus on processes that are not deductive in nature, such as probabilistic, relational^9^, or spatial reasoning^10^. Most require considerable cognitive efforts, making it difficult to understand which elementary deductive processes enter reasoning and when. If we want to understand the scope of thinking and the role of logical processes in it, we must study how logic influences the way we naturally make sense of dynamical events, which are not encoded verbally in the forms of 'premises' and 'conclusions', have no explicit deductive component, and yet trigger deductive reasoning spontaneously and effortlessly. Here, we describe the neural circuitry recruited when participants watch such dynamical scenes, tracking oculomotor reactions while they unfold. Although such scenes have no premises or conclusion, they elicit representations and inferences of logical nature in participants' minds. Our aim in the current study is to make the first steps to identify the neural correlates of elementary nonverbal deduction and their dynamics throughout a reasoning process. These are fundamental to characterize the nature of the underlying representations, which is currently unknown. Our guiding hypothesis is that reasoning can neither be reduced to a progressive construction of an analog of reality nor to its verbal counterpart alone, but that verbal and nonverbal reasoning share substantially identical representations at the level of the logical form of the thoughts they convey^11^.

### The neural correlates of verbal deduction

In contrast with elementary nonverbal deduction, considerable progress has been made towards understanding the neural bases of verbal deduction. Two facts appear clear. First, verbal reasoning is a process in which separate phases can be identified. Prominent among them stands the phase of the representation of the logical form of the premises, and the phase of the inference, when a conclusion can be reached from the premises via the application of some simplification procedure. Activations in the left inferior frontal gyrus (IFG, Broadman Area—BA44/45) and superior medial frontal (BA6/8) cortices predict when participants grasp the logical structure of the premises of problems, even when they do not draw valid conclusions^12,13^, whereas activation in the left ventro-lateral frontal (BA47) cortex predicts when they reason correctly^12^. The involvement of different brain regions suggests that the extraction of the logical form of the premises and the deductive inference rely on different processes.

Second, the neural network associated with deduction is not entirely confined within brain regions generally associated with natural language processing. On the one side, it is intriguing that the left IFG (BA 44/45) is recruited during the processing of premises, potentially indicating that this phase of reasoning exploits linguistic representations (even though caution that an overlap in location between two functions does not imply an overlap in processing^14,15^); also, the fact that such areas respond to increasingly complex syntactic constructions may indicate a role in the representation of complex logical forms^16^. On the other side, the regions engaged when logical inferences are drawn only partially overlap with the language network, even when reasoning is triggered by linguistic material. Namely, logical inference appears to involve a distributed left hemispheric network including inferior and superior frontal (BA8, BA6, BA47, left posterior BA10), and parietal areas (BA7, BA40)^4,12,13^.

Besides these insights, brain imaging studies find limited consensus to describe the neural basis of deduction, partly because of the multiple forms of reasoning and type of baseline tasks used across studies^17^, and partly because the aforementioned systematic use of language, mostly presented in written forms, makes it hard to determine whether the recruitment of linguistic areas is related to the verbal encoding of the premises^13,18^ or to the nature of logic as rooted in language^19–23^.

### Study overview

**Figure 1:**
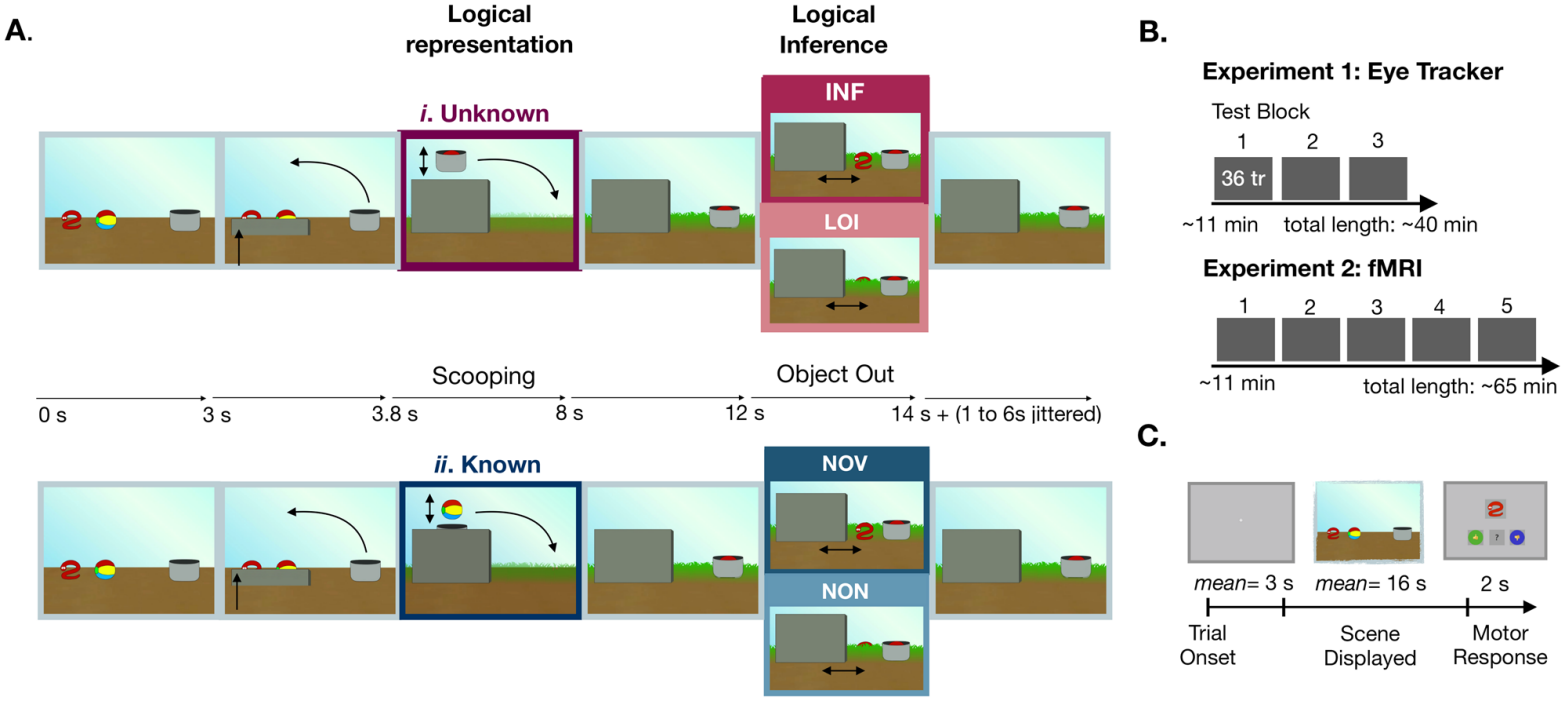
Stimuli and experimental paradigm. (**A**) Non-verbal elementary logical scenes. Two objects with identical upper parts and a cup appear on a stage. An occluder cover the objects entirely; at Scooping one object could enter the cup by being scooped from behind the occluder (*i*), leaving only its upper part visible so that its identity remains unknown (e.g., snake or ball, or an *x*); alternatively (*ii*), it could enter the cap from above the occluder, remaining visible so that its identity is known (e.g., ball). Meanwhile, a thick grass emerges and the cup moves to its original location standing still. The other object exits the occluder (Object Out) either in front of the grass (INF), thus allowing participants to infer the cup content by disjunction elimination (e.g., The snake is not inside the cup, therefore the ball is inside), or behind the grass (LOI), so that it remains unknown. The objects exit the occluder exactly in the the same way, respectively, in NOV and NON, but how they exits has no logical valence because the content of the cup is known. Finally, the object returns behind the occluder. Arrows indicate the temporal course of the scenes; timings correspond to the onset of events from A to F. A scene total length is variable, ranging from 15 to 21 s. (**B**) Design. Experiment 1 (oculomotor study) had three blocks of 36 trials each; Experiment 2 (brain imaging study) five. (**C**) Trial structure. Each trial begins with a light grey full screen followed by the display of a scene. Afterwards, in the 30% of the scenes a new trial starts immediately, whereas in the remaining 70% a response screen appears with an object in its center and participants have to decide if it matches the content of the cup (see Supplementary Material).

Here, we focus on disjunctive reasoning, a topic that has been extensively studied with verbal material^24–28^. We introduce a paradigm where elementary logical inferences can be realized entirely nonverbally, building upon recent work by Cesana-Arlotti and collaborators^29^. These authors created dynamic visual scenes in which participants could draw a conclusion about the identity of a hidden object by eliminating possibilities in accordance with logical inference. Although they were passive observers and had no logical task to perform, oculomotor markers at the moment of the inference suggested the presence of spontaneous logical processing of the scenes' content. These markers were also stable across ages, appearing in adults and in 12 and 19-month-old infants^29^, suggesting that the processes generating them are at the core of human elementary reasoning, not directly connected to language skills or language acquisition^30,31^. Following^29^, we prepared short movies where one of two objects (e.g., a snake, a ball) is first scooped by a cup (*Scooping phase*; Fig. 1A). In some cases, participants cannot know its identity (*Unknown condition*; Fig. 1A*i*), and could represent the content of the cup with a disjunction (e.g., snake OR ball) or a variable standing for an unspecified *x*; in either interpretation, this phase requires participants to create a representation containing logical elements that have to be formulated in an internal representational code. In other cases, otherwise matched for visual and temporal aspects, participants know its identity (*Known condition*; Fig. 1A*ii*) and could represent the cup content by means of a constant (e.g., ‘snake’) without the need of any further logical elements. Contrasting these two kinds of scenes at the Scooping phase can allow us to explore, for the first time to our knowledge, the neural and oculomotor signatures associated with elementary logical representations involved in the processing of dynamical scenes highly matched in their spatial and temporal parameters. The scene continues and in the *Object Out phase*, in one condition a logical inference can dissipate the ambiguity about the object in the cup (e.g., I saw a snake, so it is not in the cup; so there is a ball in the cup; *Inference* scenes; hereafter *INF*; Movie S1). In another condition, it remains unknown and no inference can be drawn (*Lack of Inference* scenes; hereafter *LOI*; Movie S3). Finally, conditions exactly matching INF and LOI conditions can be created when the content of the cup was already known, because no matter how the scene continues, no inference is needed to know it (respectively, *No Inference, Object Visibl*e, hereafter *NOV*, visually matching INF scenes, and *No Inference, Object Not Visible*, hereafter *NON*, visually matching LOI scenes; respectively, Movies S2 and S4). Neural and oculomotor markers in the Object Out phase can reveal the networks associated with an elementary logical inference, or with the maintenance of ambiguous information, compared to those where no inference occurs. We inspect the oculomotor correlates of the two phases with logical valence by studying participants’ oculomotor responses (Experiment 1) and their neural correlates in an fMRI paradigm (Experiment 2) when watching the same scenes (Fig. 1B).

## Results

### Experiment 1

#### Pupil response during the Scooping phase

**Figure 2:**
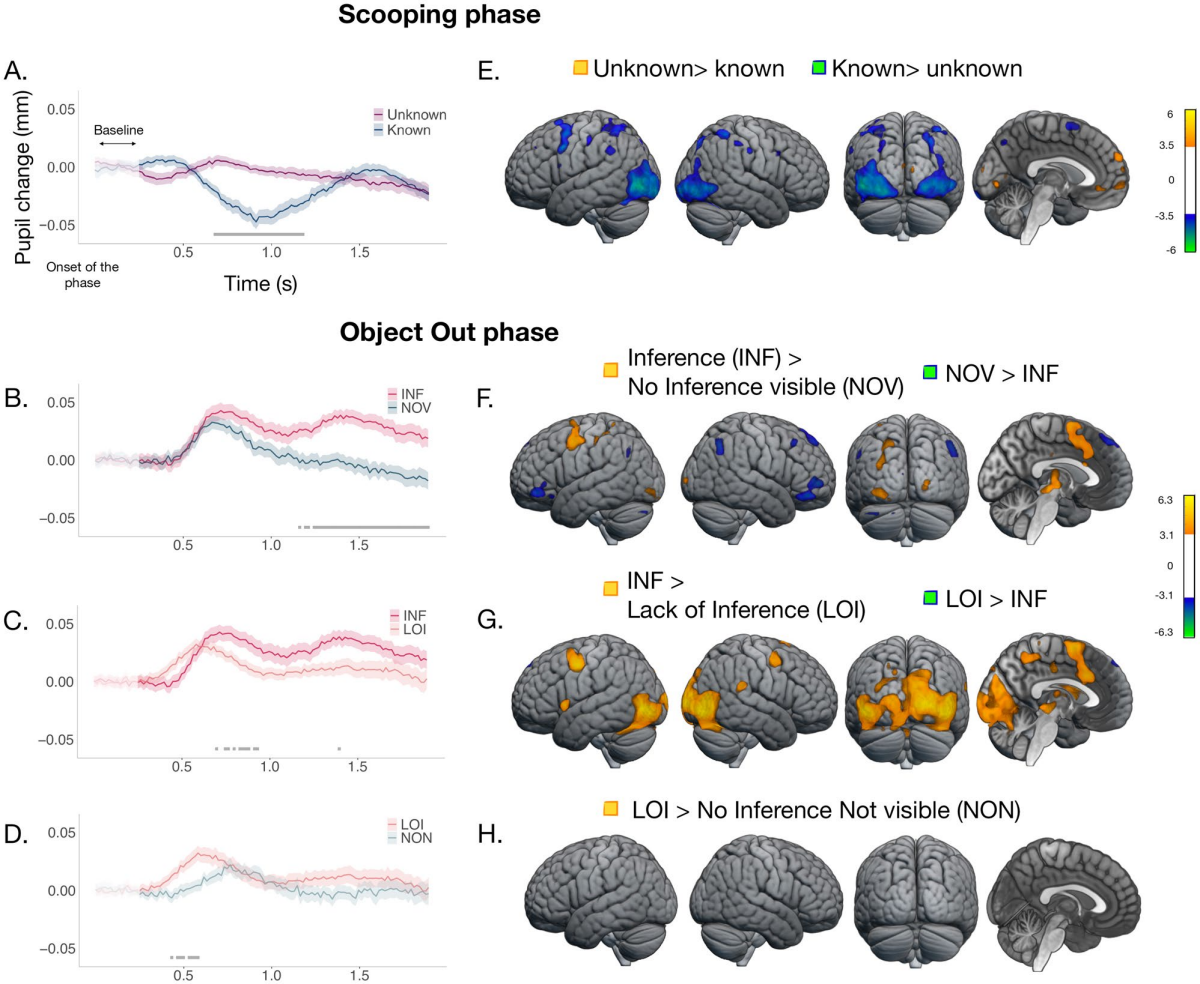
Pupil dilation (Experiment 1) and Brain activations (Experiment 2) during the Scooping and Object Out phases. At Scooping, one of two objects has entered the cup either in a visible (Known) or invisible (Unknown) manner. At Object Out, the object behind the occluder exits. Depending on the condition, the exit may be informative to deduce the identity of the object inside the cup (INF: inference needed; LOI: no inference can be drawn: NOV and NON: no inference needed). (**A–D**) Temporal course of the mean pupil dilation (SEM) from baseline. Grey horizontal lines indicate areas of effect identified by permutation tests, (**B**–**D**) additionally Bonferroni-corrected. (**A**) Unknown (purple) and Known (dark blue) conditions. Pupil size reduced in the Known compared to the Unknown condition, indicating sustained cognitive effort due to possible logical processing in the latter. (**B**) INF (red) and NOV (blue stone) conditions; (**C**) INF and LOI (light red) conditions; and (**D**) LOI and NON (light blue) conditions. Pupil dilation was higher when a logical inference was required to identify the object in the cup, or when an unknown representation had to be maintained, compared to the conditions requiring no inference (**B**, **D** respectively). Likewise, a logical inference elicited a higher pupil dilation compared to LOI, another condition with logical valence (**C**). (**E–H**) Whole brain views (lateral left and right, posterior and medial) of the main brain areas responding to the (**E**) Unknown (orange/yellow) and Known (blue/green) conditions (T > 3.47); to the INF condition (orange/yellow) contrasted to (**F**) the NOV (blue/green) and (**G**) the LOI (blue/green) conditions; and to the LOI contrasted to the NON condition (**H**) (T > 3.09). See Supplementary Table S1−S6 for other active regions.

In all the pupillometry analyses, we used permutation tests^32^ at p < 0.05 Family-Wise Error Rate (FWER) corrected, complemented with analyses with the Guthrie-Buchwald (G-B) method aimed at identifying the uninterrupted temporal regions of the effects^29,33^. We first checked whether participants' pupil responses could signal differences in the processing of a logical representation (‘Snake or ball inside the cup’, or ‘unknown* x*’) compared to the representation of a known object. We contrasted the Unknown and Known conditions pooling INF and LOI against NOV and NON trials (Fig. 2A). The pupil diameter reduced in the Known condition compared to the Unknown condition, where it remained dilated across the window of interest. This effect appeared in a temporal window extending from about 0.7 s after the onset of the phase to about 1.2 s (*p* < 0.05, max Cohen's *d* = 0.35, Fig. 2A. G–B results were consistent with the permutation tests, reported in Supplementary Materials).

#### Pupil response during the Object Out phase

We then explored the pupil response when conclusions can be drawn. We first examined whether a situation requiring an inference by exclusion elicited a different response compared to a situation not requiring it. Pupil diameter was higher in the INF than the NOV condition (Fig. 2B; at Object Out, p-values were additionally Bonferroni-corrected for multiple comparisons; *n* = 2). This effect extended from around 1.1 s after the onset of the phase until 1.9 s (max Cohen's *d* = 0.28). The analysis confirms and extends the results reported in^29^. We then compared the two other visually identical conditions having, or lacking, logical valence: LOI and NON. In both, no conclusion can be drawn, but LOI requires the sustained representation of something unknown, while NON requires the simple representation of a known object. The representation of the unknown remains distinct from that of a known object and comes with an additional cost, appearing from 0.4 to 0.6 s from the onset of the phase (max Cohen's *d* = 0.29; Fig. 2D). Thus, both conditions with logical valence are processed differently from their visually matched known conditions. Finally, we ran exploratory analyses on two further pairs of conditions. First, we compared INF, where an inference can be drawn, to LOI where an inference cannot be drawn but the representation of an unknown object has to be maintained. Adults' pupil dilated more in the INF than the LOI scenes, from about 0.7 after the onset of the phase until 1 s and at other later points (max Cohen's *d* = 0.18; Fig. 2C), suggesting that the cost of making an inference is higher than that of maintaining the representation of an unknown object. Second, we compared NOV and NON, which have visual properties identical to INF and LOI but require no logical processing because the object in the cup is known. Oculomotor responses differed only earlier, between 550 and 650 ms, likely responding to the initial visual differences between conditions, and they quickly became indistinguishable (Supplementary Fig. S2A), suggesting that the effect found comparing INF and LOI could be due to the cost of inference making over and above such differences (see Supplementary Fig. S3 for a unitary presentation of all the conditions). However, because the interaction between these couples of conditions was not significant (Supplementary Fig. S4), no firm conclusion about how differences among logical representations impact pupil dilation at Object Out can be drawn.

### Experiment 2

#### Brain activation during the Scooping phase

The Unknown condition produced stronger activations than the Known condition in the medial orbitofrontal cortex (BA10, BA11), medial prefrontal gyrus (mPFC; BA10), in the left middle temporal pole (BA38) and dorsal occipital cortex (BA19, BA18). The Known condition produced instead stronger activations in the inferior occipital (BA18, BA19), the right inferior temporal (BA37), the left inferior parietal (BA40 extended into BA7) cortices, the left precentral gyrus [PrC(BA6)], the right superior frontal (BA6) cortex, and left inferior opercularis (BA44) (Fig. 2E and Supplementary Tables S1–S2).

#### Brain activations during the Object Out phase

The contrast between the INF and NOV conditions, which are visually identical in the Object Out phase (see Fig. 1A), revealed an extensive set of areas mainly involving the left PrC (BA6; extended into the supplementary motor area), the left parietal (BA40, BA7), and the occipital (BA17, BA18, BA19) extended into the temporal (BA37) cortices. All these areas were more active when the scenes licensed an inference (Fig. 2F; Supplementary Table S3). Conversely, the NOV condition produced stronger activations than the INF condition in the right frontal (BA46, BA47), medial frontal cortex (BA9, BA8), and in the left inferior frontal gyrus (BA47, BA45). Unlike the oculomotor data, the brain response comparing the visually identical conditions when no inference was possible (LOI) and no inference was needed (NON) did not yield significant differences (Fig. 2H).

Finally, we contrasted the INF and LOI conditions, where, respectively, the object exiting the occluder triggers the inference about the identity of the object in the cup, or its identity remains unknown. Regions within the occipital cortex, the left superior motor area (BA6), the PrC (BA6), and the left parietal cortex (BA40, BA7), were more active in the INF condition. The LOI condition was more active than the INF in a single cluster located in the left superior middle frontal gyrus (BA9; Fig. 2G; Supplementary Tables S5–S6). By contrast, NOV and NON, the two conditions where the object inside the cup was known by visual perception, did not reveal any difference (Supplementary Fig. S2B). We also explored the interaction (INF > LOI) > (NOV > NON): being essentially driven by the INF > LOI contrast, it reveals the same regions (Supplementary Fig. S5, Supplementary Tables S7–S8).

#### ROI analyses of verbal reasoning and language areas

**Figure 3:**
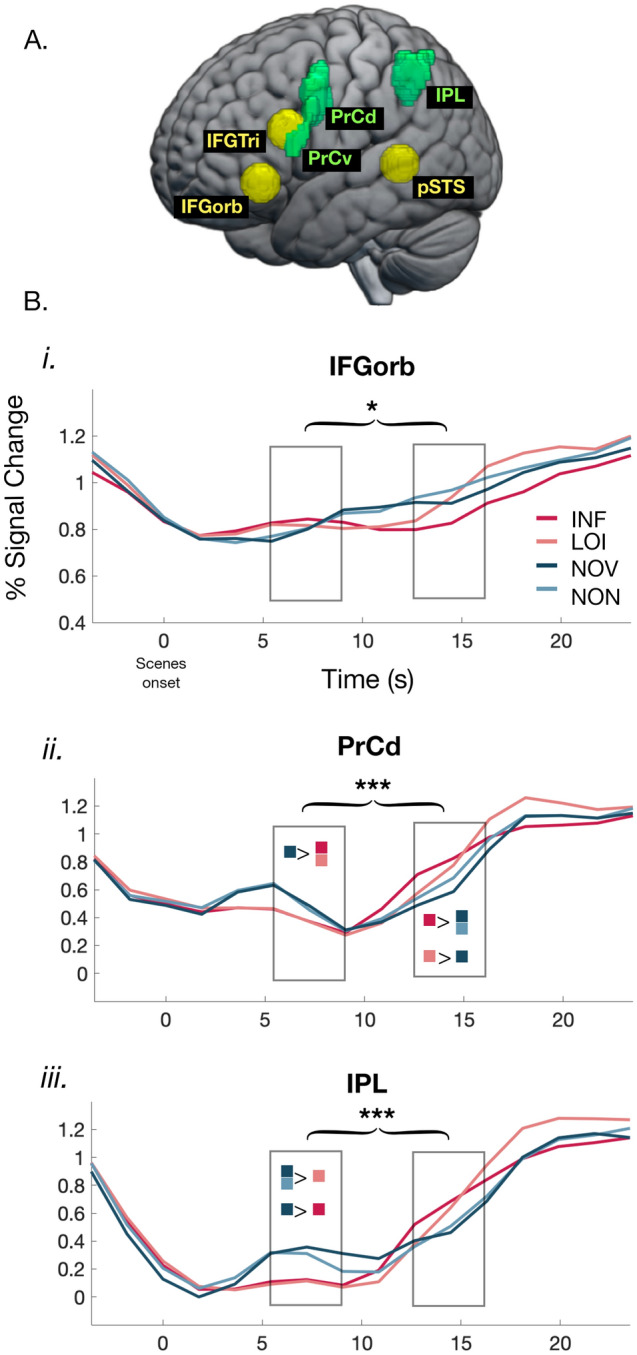
Temporal course of brain activations in Regions of interest (ROIs). (**A**) ROIs associated to syntactic processing, from (14) (IFGorb- inferior frontal gyrus orbitalis; IFGtri- inferior frontal gyrus triangularis; pSTS- posterior Superior Temporal Sulcus, in yellow), and to verbal reasoning from (5) (*PrCd*—precentral dorsal, *PrCv*—precentral ventral—colored in green; IPL, inferior parietal lobule). (**B**) Signal changes in ROIs with interactions between Phase and Condition (see Supplementary Materials for all the ROIs). Graphs represent mean coefficients per ROI computed from a FIR model; x-axis extends from Trial onset to the Response phase included (total length: 28 s). Rectangular boxes indicate the Scooping and Object Out phases. Colored squares indicate differences between Conditions, and asterisks spanning across phases interactions (Bonferroni corrected, N = 6), resulting by separate ANOVAs for each ROI.

To assess the role of regions identified in previous works associated with verbal logical reasoning^5,34^ and syntactic processing^16^, we ran a region-of-interest (ROI) analysis (Supplementary Materials). The ROIs were generated based on the results reported in previous works, thus removing any danger of circular analysis. We selected regions reported as active when the elementary logical connectives are represented [left posterior inferior frontal gyrus (pIFG)]; when deductive inferences are triggered [left precentral (PrC) dorsal and ventral, and inferior parietal (IPL)]; and when sentence forms of different constituent complexity are processed [posterior superior temporal sulcus (pSTS); inferior frontal gyrus orbitalis (IFGorb; because IFGorb (BA47) in^16^ overlapped with pIFG in^34^, we refer to the coordinates reported in^16^); and pars triangularis (IFGtri)] (Fig. 3A). Our main interest was to explore changes in the functional role of these areas during the different moments of a reasoning process.

We performed separate repeated measures ANOVAs in each ROI based on the activation levels estimated in a Finite Impulse Response (FIR) model, with Condition (INF, NOV, LOI, NON) and Phase (Scooping and Object Out) as within-participant factors and participants as a random factor. The rationale for choosing the FIR model is that it better estimates the temporal unfolding of the signal in the specific ROIs. Since results from both the HRF (used for the whole brain analysis) and FIR models were equivalent (Supplementary Table S9) we report here the activations from the FIR model. We detected an interaction between Phase and Condition in IFGorb (F(1,57) = 4.54; *p* = 0.037; Fig. 3B*i*), PrC dorsal (F(1,57) = 20.9;* p* < 0.0001; Fig. 3B*ii*), and IPL (F(1,57) = 11.9; *p* < 0.001; Fig. 3B*iii*; Bonferroni corrected for multiple comparisons). The nature of the interaction was not the same in these areas. In PrC dorsal and IPL, post-hoc analyses revealed that, at Scooping, conditions in which the object was known elicited higher activation compared to those in which the object was unknown (in PrC dorsal: NOV > INF *p* = 0.03; NOV > LOI, *p* = 0.03; in IPL: NOV > INF, *p* = 0.03; NOV > LOI, *p* = 0.01; NON > LOI,* p* = 0.04; Bonferroni corrected). At Object Out, an opposite trend appeared: conditions not requiring any logical processing were less active—in PrC dorsal NOV and NON were less active than INF (respectively, *p* < 0.0001 and *p* = 0.01), and NOV was less active than LOI (*p* = 0.04), even if the visible stimulus in the NOV condition was richer. IPL displayed the same profile, although differences between conditions were not significant at Object Out. In contrast with these areas, in IFGorb activity tended to increase from Scooping to Object Out for those conditions in which the object was known (*p* = 0.06 for NOV and *p* = 0.009 for NON): no differences emerged at Scooping, but at Object Out there was a strong tendency for this area to be more active in the NON than in the INF condition (*p* = 0.058). No effects were found in IFGTri, pSTS and PrC ventral after Bonferroni correction (for a complete report on ROIs see Supplementary Fig. S6; Supplementary Table S9).

#### Lateralization of reasoning areas

We evaluated lateralization of the activity found within the ROIs comparing them with their right hemisphere mirrors. Three-way ANOVAs for each ROI with Phase, Condition and Hemisphere as within-participant factors revealed no main effect of lateralization, except in PrC dorsal. In that area, there was an overall stronger right-lateralized activity (F(1,19) = 12.04, *p* = 0.015), which, however, was modulated by Phase and Condition. It increased from Scooping to Object Out only in the left hemisphere, as revealed by a double interaction between Hemisphere and Phase (F(1,19) = 25.59, *p* = 0.0004) and the relevant post-hoc analyses (left hemisphere: p < 0.00001; right: *p* = 0.6). More importantly, there was a triple interaction between Hemisphere, Condition and Phase (F(3,57) = 12.9, *p* < 0.0001). Post-hoc analyses showed that activity increased from Scooping to Object Out in the left hemisphere only for the logically relevant conditions (INF and LOI), relative to the conditions which were logically irrelevant. Notably, INF/LOI were less active than NOV/NON at Scooping, but were more active than NOV at Object Out; in this phase, generally the response for fully visible objects was inferior to that for partially occluded objects. By contrast, at Object Out the right hemisphere did not respond to differences between conditions (Supplementary Table S11). A similar but less marked profile appeared in IPL, where a triple interaction between conditions was detected (F(3,57) = 5.5; *p* < 0.01). Post-hoc analyses showed that at Scooping activity in both hemispheres was higher for NOV/NON relative to INF/LOI (that is, for the Known conditions relative to the Unknown conditions), but at Object Out the right hemisphere did not record differences between conditions, whereas the left hemisphere registered more activity for the INF than the NOV and NON conditions (Supplementary Table S12). These results suggest that the involvement of such areas in the phases requiring logical operations (an inference, or the maintained representation of an unknown when no inference can be drawn) was particularly pronounced in, or specific to, the left hemisphere. The presence of the triple interaction, together with the fact that the design made it impossible to prepare a specific finger response before the presentation of the response screen, lead us to exclude that the increase in left hemisphere activity at Object Out was due to motor response preparation. Besides PrC dorsal, double interactions between Hemisphere and Phase also appeared in IfGorb, IFGtri, IPL and PrC ventral. In all cases, these interactions were due to the fact that at Scooping the right hemisphere was more active, but only at Object Out did the activity increase in the left hemisphere.

#### Relations with MDN and DMN

The areas we reported in the whole brain analyses partially overlap with the Default Mode (DMN) and the Multiple Demand (MDN) networks, which thus could have a role in our result. To evaluate whether these networks are involved in specific conditions of our task, we ran uncorrected ANOVAs in the regions composing the two networks (Supplementary Materials; Supplementary Tables S13–S14; Supplementary Figs. S8–S9). Overall, only the 32% of the voxels in the DMN responded to our task even by this lenient criterion. Some areas also had mutually conflicting activation profiles, suggesting that the activity of the DMN network is unrelated to our results. As for MDN, the areas we report active at Object Out do partially overlap with it, with, activities coherent with our results. Nevertheless, only the 40% of the MDN voxels responded, while most of the network was silent. Because elementary logical reasoning is likely to be involved in most, if not all, cognitive tasks, this overlap is not surprising, but because it only affects a subset of the network, activity in MDN cannot account for the overall pattern we report. Involved in many tasks of radically different nature^35^, MDN is thus of little use to identify the neural correlates of operations as specific as the elementary deductive steps we focused on. Rather than being explained by MDN activity, we see the current results as a step towards the understanding of one of the fundamental processes involved in the disparate list of tasks attributed to MDN.

#### Activations during response preparation

Finally, we explored an unexpected effect that appeared at the selected ROIs in the temporal course of the hemodynamic response, when participants had to plan their answer to the task. Exploratory ANOVAs at each ROI with the same design as those described above revealed that activation was higher for the LOI than in the INF condition in PrC dorsal (*p* = 0.02), IFGorb (*p* = 0.019) and IFGtri (*p* = 0.01; Supplementary Materials). This particular and diffused peak of activity when the identity of an object cannot be determined suggests that a logical representation of an object in a scene (as an unknown x or as a disjunction of possibilities) persists over time and can be reactivated upon task demands.

## Discussion

With a novel methodology, we began studying the dynamics of nonverbal elementary logical deduction, identifying the oculomotor reactions and the neural circuitry involved in creating logical representations and making inferences from dynamical visual scenes. Participants had to generate their own representations by watching them, in some cases making a logical deduction to determine the identity of a hidden object. Our paradigm eschews linguistic content and the forced segmentation induced by the presentation of explicit premises, present in most studies about logical reasoning. Without any explicit logical task, participants spontaneously parsed the dynamical scenes in temporal phases with different logical status: an initial representation of the structure of a situation, and a successive phase in which deductions could be drawn. These phases are associated to different patterns of brain activity and display characteristic oculomotor markers. In particular, beyond occipital activations induced by visual processing, the representation of an unknown object recruits the medial prefrontal cortex (mPFC, BA10). Involved in many cognitive functions, mPFC has also been related to general inferences during text comprehension^36^, in planning, decision making, and control of alternative strategies^37,38^, and during hierarchical processing in natural language^16^. Interestingly, lesions in medial frontal areas impair the monitoring of a logical reasoning also when presented verbally^39^. The mPFC activity we found is not linked to a generalized involvement of DMN.

These consideration suggests a role for mPFC in the representation of an unknown object, and strengthen the case for its core role in logical reasoning, perhaps in monitoring the construction of a space of alternatives (‘*x* is A or *x* is B’)^31^. Oculomotor data further support these conclusions. At Scooping the pupil is less dilated when an object identity is known relative to an unknown condition, a fact that low-level visual differences alone cannot explain (see Supplementary Materials, §1, Data processing and Analysis, *ii*, Supplementary Fig. S7). Overall, the results indicate that processes involved in the setup of a logical scaffolding of the environment are ongoing even when the simplest logical representation of a scene is constructed, regardless of whether it is induced by direct experience, as in our paradigm, or by a verbal description of it, as in previous studies. Thus, the conjoined neural and oculomotor evidence indicates that constructing the representation of an unknown* x*, or of a disjunction, from a dynamic scene is qualitatively different from the representation of a simple object.

Compared to when logical representations are generated (Scooping), the Object Out phase (where the logical inference can eliminate alternative possibilities) displays a different oculomotor and neural profile. Oculomotor markers reveal cognitive activity engendered by logical processing: the pupil is more dilated when an inference is needed compared to identical scenes that require no deduction. A deduction is also associated with a pattern of activation mostly located in the frontal and parietal areas. Remarkably, the overall fronto-parietal activity in whole brain analyses after an inference resembles that of a known object before an inference.

ROI analyses confirm that the dorsal PrC and the IPL regions, which mark object individuation at Scooping, tend to change activity pattern at Object Out, becoming more active in the Inference condition (INF) and, less markedly, in the Lack of Inference condition (LOI) compared to their controls. To understand why, consider that in our task the role of inference is to determine object identity. After all, knowing the identity of an object by perceptual information (as in the Known condition in the Scooping phase) or knowing it via inference (as in the Object Out phase, when the scene licenses the inference) must result in the same final mental representation: the object in the cup. What changes is the process by which such a representation is created: in the former case it is generated from observation, while in the latter case it is mediated by reasoning.

Intriguingly, whole brain analyses at Object Out revealed activity in a reasoning network highly compatible with regions involved in elementary verbal reasoning, particularly in the lateral parietal (BA7, BA40) and in precentral cortices (BA6)^5,12,21,34,40–42^. The considerable accord in neural activity between verbal reasoning and the Object Out phase of our task indicates the centrality of such areas in any reasoning process, regardless of the modality of stimulus presentation, suggesting that in all forms of reasoning, whether verbally-induced or induced by the observation of a real-life situation, deduction involves the recruitment of abstract logical forms from which conclusions can be drawn. Reasoning in the brain operates at a level of abstraction without substantial difference between the verbal and the nonverbal.

### Comparison with the "core deduction network"

In a series of important papers, Monti and his collaborators argued that the ventrolateral frontal cortex (posterior BA10/anterior BA47) is part of a "core" deduction network^13,18^, responsible for transforming logical structures and "substituting or eliminating logical connectives" [^4^, pp. 1010–1011]. We found that mPFC (or medial BA10) [− 1.5 61.5 − 3], an area that does not overlap with the coordinates reported in^4^ [− 48 46 − 2], is active when a logical representation has to be created (Scooping), but not when a deduction needs to be drawn (Object Out) (Supplementary Materials, Supplementary Table S10). Thus mPFC may have a more focused role than that assumed by Monti and collaborators. We do find activation in BA47 [− 42.5 39 − 10] in an area that may overlap with it. Still its activity modulates in a way difficult to square with a core role in deduction proper: BA47 at Object Out is even more active for the non-logical (NOV) than for the inference condition (INF), an activation starting apparently very soon after the Scooping phase. Such a pattern seems more compatible with a role in memorizing the known information and protecting it from interference from the evolving scene. This possible interpretation is supported by the FIR analyses, which show a very early increase in activation of BA47 in the NOV and NON conditions, prior to and unrelated to deduction (Fig. 3B*i*; Supplementary Materials).

A closer comparison with Monti's studies is anyway difficult. Critical aspects of our paradigm are different; our scenes can distinguish phases of reasoning, separating logical representation from inference, whereas theirs and other studies conflate the two phases, thus making it impossible to describe modulations of the functional role of the networks across the reasoning process. When a problem is presented verbally and left available on-screen during the whole trial, participants may repeatedly process its logical form, thus potentially generating an activation not necessarily related to deductive inference. Furthermore, the design of the verbal reasoning experiments by Monti and collaborators is based on the assumption that complexity affects the deductive machinery, suggesting that its activity depends on the complexity of a problem overall^18^, a factor we did not manipulate. Most importantly, studies based on verbal problems present the logical forms upon which a reasoning process must operate by providing the premises, whereas in our task participants have to extract the relevant aspects of a fleeting scene, quickly coding and representing its gist while it unfolds. The need to generate this representation can explain the prominent role of mPFC and the absence of any fundamental role of IFGorb (BA47) in our nonverbal deduction. The proper evaluation of these hypotheses will require further research that more closely compares verbal and non-verbal reasoning, and the role of complexity in it. In light of the current results, it is difficult to attribute a core role to BA47 in elementary deduction, at least when participants must reason based on direct visual stimuli not involving the processing of problems formulated in language.

### Limitations and further developments

While we believe that our results open novel perspectives on our understanding of the role of deduction in cognition, our study has several limitations that further research should address. Even if no logical task is given, and hence participants spontaneously extract the representations on which they reason from the scenes, the study does not allow to assess the role of language areas in a within-subject design. The individual localization of language areas for every participant would allow for a better evaluation of their role in the different moments of nonverbal elementary deductions. Further explorations with concurrent verbal tasks could also probe the ability to carry to completion elementary deductions even under load of language resources. The data also point at differences between kinds of logical representations, although our task was less sensitive to them. While both neuroimaging and oculomotor data show that conditions with logical valence were always different from the visually matched conditions without logical content (INF vs. NOV and LOI vs. NON), our task captures differences between the sustained representation of an unknown object (LOI) and the application of a rule of inference (INF) in the whole brain analysis, but only marginally in the oculomotor data. Further studies are needed to address how task demands modulate the signal strength of logical representations across time. Finally, while our data show that the representation of an unknown object is associated to characteristic oculomotor and brain activity responses distinct from those elicited by a known object, the exact nature of the representation of the unknown—whether in the form of a disjunction of individual alternative possibilities, or as a variable ranging over a given domain, is still to be determined.

## Conclusion

Overall, our data support the existence of an organized network that represents situations with logical content and realizes elementary logical inferences by simply observing scenes. Remarkably this network is involved not only when people generate a logical inference, as it might be expected, but also when they generate a structured logical representation from visual observations. In the deduction phase, but not when a logical representation is generated, the observed network closely resembles that involved in verbal reasoning, as if both visual and verbal material elicited a common level of representation and underwent similar computations. The current data are compatible with the possibility that the overlap of activity in verbal and nonverbal reasoning occurs, not because reasoning unfolds in natural language^43,44^, but rather because logical forms are represented and acted upon via rules of derivation regardless of the vehicle—whether linguistic or not—that triggers a reasoning process^11^. In short, it's not that reasoning is linguistic; rather, it is language that is much more dependent on logical processes than generally recognized^45^. The evidence shown here contributes to clarifying the neural mechanisms of elementary logical reasoning in the absence of language. Here we simply explored the tip of the iceberg. Deductive reasoning, verbal or nonverbal, goes way beyond simple disjunction elimination. With a simple paradigm, we showed how to provide information about its nature and temporal course. Extending it to other forms of situation-induced inferences will allow us to better understand the nature of human thought.

## Materials and methods

### Participants

Twenty-eight adults (20 females; mean age = 23.41; range age = 18:48) were retained for Experiment 1. Eight additional adults were tested and excluded from the analysis due to poor oculomotor data quality. All of them were recruited through the Center for Brain and Cognition database at Universitat Pompeu Fabra (Spain), where the experiment took place. Another group of twenty adults (12 females; mean age = 24.57; range age = 18:36) was scanned for Experiment 2. They were recruited through the Cognitive Neuroimaging Unit database at NeuroSpin (France), where the acquisitions were performed. All participants gave written informed consent and received compensation for their participation. These studies were approved by the regional ethical committee Parc Salut del Mar. All experiments were carried out in accordance with relevant guidelines and regulations.

### Stimuli

The stimuli were animations generated with the Apple Keynote as QuickTime movies, adjusted for timing, compiled at 60 fps and compressed with Apple Intermediate Codec format. Three objects with identical top parts (a snake, a ball, and an umbrella) could be combined into three possible couples (Supplementary Fig. S1). This arrangement forced participants to keep track of the couple presented in each trial right from its beginning. The stimuli consisted of 36 unique scenes. They resulted from the combination of (1) object couple (snake-ball, snake-umbrella, ball-umbrella); (2) spatial arrangement of objects onstage (left, right); (3) location of the scooped object relative to the cup (near, far); and (4) experimental conditions (12 inference (INF), 12 lack of inference (LOI) and 12 No Inference (6 NOV and 6 NON). Scenes lasted from 15 to 21 s. They were composed of an initial phase (3 s); the Scooping phase (5 s, Fig. 1A); a steady phase (4 s) to allow for the hemodynamic response to stabilize; the Object Out phase (2 s); and a final steady phase lasting 2 s on average, ranging randomly between 1 to 6 s with an exponential distribution. The two phases a priori selected for analysis were matched for several parameters across conditions. For the Scooping phase, the timing and movements were matched so as to minimize visual differences between the Unknown and Known conditions. For the Object Out phase, the two conditions when the object exiting the occluder was visible (INF and NOV) were identical, as well as the two conditions when it was not visible (LOI and NON). In all conditions, the duration of the phases was identical. At the end of a trial, participants could see an object on screen. They had to report if this was the object inside the cup. Notice that this task is completely unrelated to logical reasoning, and the instructions were never mentioning, either explicitly or implicitly, reasoning or deduction. The object was presented in the upper center on the screen. Below, images appeared for choice selection (a thumb up for ‘yes’, a question mark for ‘I cannot know’, and a thumb down for ‘no’). The object could be either one of those composing the couple (expected answer: thumb up), or the third object not presented in the trial (expected answer: thumb down), or else the top part of the object alone, in which case the answer could not be given (expected answer: question mark). These features of the task forbade participants to prepare a specific response prior to the presentation of the response screen.

### Procedure

Each trial began with a light grey full screen followed by the display of the scene and ended with the presentation of the response display in the 70% of the trials (Fig. 1C). Each block contained twelve trials of the INF and LOI conditions, and six trials of the NOV and NON type. The 36 trials of each block were presented pseudorandomly in a sequence respecting the following constraints: (a) at most three consecutive scenes of the same condition; (b) at most six consecutive scenes requiring an answer; (c) at most three consecutive scenes with the same couple; and, (d) at most four consecutive question trials requiring the same response. For those trials in which a response was required, participants could respond with a button box using their thumb, index and middle fingers. Each block lasted around 13 min. Experiment 1 contained three blocks and Experiment 2 five (Fig. 1B). Participants were given a self-timed break between blocks.

### Eye data acquisition and analysis

See Supplementary Materials for details.

### fMRI data acquisition and analysis

See Supplementary Materials for details.

## Supplementary Information

Supplementary Information 1.

Supplementary Video 1.

Supplementary Video 2.

Supplementary Video 3.

Supplementary Video 4.

Supplementary Information 2.

## Data Availability

Data and analysis scripts for Experiment 1 are available at https://osf.io/bp8wu/?view_only=5057a0c019244dfd838d1a0d1f432970. Data for Experiment 2 are available upon request. Point of contact: anamartinsalguero@icloud.com.
