# Supplementary material for: Seeing inferences: brain dynamics and oculomotor signatures of non-verbal deduction
Source: Sci Rep. 2023 Feb 9;13:2341. doi: 10.1038/s41598-023-29307-3 (PMC9911777; doi:10.1038/s41598-023-29307-3)
Supplement: Supplementary file 1 — Supplementary Information 1. [file 41598_2023_29307_MOESM1_ESM.pdf]

Supplementary Information for

Seeing Inferences: Brain dynamics and oculomotor signatures of non-verbal deductions

Ana Martín Salguero *[§&], Carlo Reverberi *[†], Aldo Solari [‡], Luca Filippin [§], Christophe Pallier *[¶], and Luca L. Bonatti *[§#]

[§] *Center for Brain and Cognition, Universitat Pompeu Fabra, Ramon Trias Fargas, 25-27, 08005 Barcelona, Spain*

[†] *NeuroMI-Milan Center for Neuroscience and Departments of Psychology, University of Milano–Bicocca, 20126 Milan, Italy*

[‡] *Department of Economics, Management and Statistics, University of Milano–Bicocca, 20126 Milan, Italy*

[¶] *Cognitive Neuroimaging Unit U992, Institut National de la Santé et de la Recherche Médicale, Commissariat à l'Énergie Atomique et aux Énergies Alternatives, Direction de la Recherche Fondamentale/ Institut Joliot, Centre National de la Recherche Scientifique ERL9003, NeuroSpin Center, Université Paris-Saclay, 91191 Gif-sur-Yvette, France*

[&] *Institut Jean Nicod, Département d'études cognitives, Ecole Normale Supérieure, EHESS, CNRS, PSL University, 29 rue d'Ulm, 75005 Paris, France*

[#] *ICREA, Ps. Lluís Companys 23, 08010, Barcelona, Spain*

Supplementary text

1. Pupil dilation data and analyses for Experiment 1
2. Imaging data and analyses for Experiment 2
3. Regions of Interest and response profiles for Experiment 2
4. Exploratory analysis of the MPFC activity found at the Scooping phase of Experiment 2
5. Exploratory analysis of the Default Mode and Multiple Demand Networks
6. Exploratory analysis on the posterior BA10 / anterior BA47 reported in [1] for Experiment 2

Appendix 1: Participants' Instructions for Experiment 1

List of Figures

Figure S1 Object pairs used in the experimental task

Figure S2 Pupil dilation (Experiment 1) and Brain activations (Experiment 2) during the Object Out phase

Figure S3 Pupil dilation in all conditions of Experiment 1 during the Object Out phase

Figure S4 Pupil dilation differences in the logical and nonlogical conditions of Experiment 1 during the Object Out phase

Figure S5 Brain activations during the Object Out phase for the interaction contrast (INF>LOI)>(NOV>NON)

Figure S6 Brain activations in Regions-of-interest

Figure S7 Pupil temporal course during the Scooping in Cesana-Arlotti et al. (2018)

Figure S8 Region-of-Interest Analysis on the Default Mode Network

Figure S9 Region-of-Interest Analysis on the Multiple Demand Network

List of Tables

Table S1-S8 Regions with effects in the whole-brain analyses

Table S9 Regions-of-interest (ROI) post-hoc analysis

Table S10 Post-hoc comparisons for the interaction between Condition and Phase found in the prefrontal area (BA10)

Table S11 Post-hoc comparisons for the triple interaction found in ROI PrC dorsal provided by Reverberi et al. (2007)

Table S12 Post-hoc comparisons for the triple interaction found in IPL provided by Reverberi et al. (2007)

Table S13 Regions of the Default Mode Network with interaction effects

Table S14 Regions of the Multiple Demand Network with effects of Condition at Object Out

1. Pupil dilation data and analyses for Experiment 1

Procedure

The experiment was run in a lighted room. Participants sat at 60 cm away from the eye tracker and from the monitor that displayed the scenes. A wireless keyboard (Apple Magic Keyboard) placed in front of them was used to collect responses, with the H, J, and K keys mapped onto to the multiple-choice panel displayed at the end of each scene. Before the experiment started, we instructed participants to be attentive to the whole movie sequence because, after some movies, an additional display would appear for them to indicate if the object presented in the center of the screen corresponded to the object inside the cup. After that, participants watched some movie examples so as to remove potential novelty effects during the test. Then, participants underwent a nine-point calibration procedure. Finally, the experiment started. Eye data were recorded at 60 Hz with a Tobii XL60 eye tracker. The experiment was controlled by an Apple Mac Pro connected to an external monitor for experimental control and to the eye tracker for stimuli presentation and data collection. PsyScope X, build 88 (http://psy.cns.sissa.it/), controlled the experiment.

Data processing and Analysis

(i) Temporal analysis

Pupil dilation was considered for analysis if data from both eyes at each sample were valid. Pupil was processed in the two temporal windows of interest (Scooping and Object Out, 2 s each). To identify artifacts, we first smoothed the data with standard algorithms (locally weighted linear regression with a span of 30%). We then computed the residuals of the the smoothed data per trial and excluded the points exceeding 3 sd of the residuals . After outlier elimination, only trials with had both 70% of valid samples in the windows of interest and 70% of valid samples in the respective baseline windows were considered for the analysis. Pupil was then baseline-corrected by subtracting the average dilation value across the first 18 samples (300ms). Participants with less than one-third of valid trials according to the above criteria were also excluded from analysis (n=6).

To test for temporal differences between conditions, univariate analyses were carried out under a permutation framework [2] at the Scooping and the Object Out phases separately. At Scooping, we performed a one-tail Wilcoxon test comparing the average pupil dilation in the Unknown and Known conditions at each temporal sample. At the Object Out phase, we performed a one-tail Wilcoxon test among the main conditions of interest INF>NOV and LOI>NON. We also performed exploratory analysis comparing the two logically relevant (INF and NOI) and the two control conditions (NOV and NON). Permutations were ran over the average time course per condition per participant, using Signed Rank tests. 30.000 permutations were ran and the reported p-values were Family Wise Error (FWE) adjusted. Permutation were performed using the R package flip (Finos, L. (2018). "Multivariate Permutation Tests'; https://cran.r-project.org/web/packages/flip/index.html).

For the two planned pairwise comparisons at the Object Out phase (INF vs. NOV; LOI vs NON), we additionally corrected the obtained p-values under FWE correction by the number of paired comparisons (2, Bonferroni). Permutation results are reported in the main text. For the exploratory comparisons (INF vs LOI; NOV vs NON) and their interaction (INF-LOI)-(NOV-NON), we only applied the FWE correction.

Guthrie and Buchwald (G-B) method [3] was also computed to identify uninterrupted temporal regions with differences among conditions. They are reported here for ease of comparisons with previous works [4]. We generated 30,000 time series from the average time course per participant per condition. The computation was performed using the R package ERP (https://cran.rstudio.com/web/packages/ERP/index.html). The package computes the sequences whose lengths are too large relative to the null distribution of significant run lengths, assuming a lag-1 autoregressive correlation model, with a type-I error level of 0.05. To these results, we applied the same corrections as for the permutation analyses reported above. In the Scooping phase, this analysis identified an uninterrupted temporal region in which the Unknown and Known

conditions differed, spanning from about 0.6 s after the onset of the phase to about 1.2 s. This region overlaps with the window of effect identified by the permutation method.

At Object Out, comparing INF and NOV, we found a continuous region of differences from 1.1 to 2 s from the onset of the phase, an area in accord with the permutation method. Comparing LOI and NON an - uninterrupted temporal region, from 0.4 to 0.6 was detected, encompassing the significant points found with the permutation test. For the INF and LOI. G-B test revealed three continuous temporal regions of differences extending from 0.4 to 0.55 s, from 0.7 to 1.05s and from 1.3 to 1.5s, a considerably larger superset of the interval identified by the permutation test. Lastly, in the NOV-NON contrast a continuous time interval between 0.55 and 0.71 s was detected, encompassing the sporadic time points identified by the permutations. Thus, overall the G-B method confirms and extends the effects reported in all the comparisons both at Scooping and Object Out.

(ii) Exploring the role of visual differences at Scooping: Temporal analysis of Experiment 7 in [4].

Our scenes are generally matched for visual and temporal structures but contain a visual difference between the Known and Unknown conditions at the beginning of the Scooping phase. In the Known scenes, an object moved up and down before entering the cup. In the Unknown scenes, the cup lowered down hidden by the occluder, then with the object inside exited and performed the same movements as those performed by the object in the Unknown condition. While we deem it unlikely, pupil dilation during the Scooping phase could have been affected by these differences . To evaluate this possibility, we reanalyzed the pupil dilation data gathered in [4], Experiment 7. In that experiment, during the Scooping phase Known and Unknown conditions exactly match in visual features. This is obtained by realizing the Known condition showing the identity of the object in the cup before the Scooping phase (Fig. S4, *A*), at the beginning of the scenes. Although the time available for analysis in this phase of the experiment are not optimal (1.1 s), we explored whether the same pupil dilation difference found in Experiment 1 of the current paper was present as well (Fig. 2). We preprocessed and analyzed these data using the same criteria described above. Permutation tests showed that that the Unknown condition elicited a higher pupil dilation than the Known condition, despite the identity in visual properties, from about 800 to 1060 ms since the onset of the phase (Fig. S4, *B*). This concordance in oculomotor reaction suggests that the difference in pupil dilation between the Unknown and Known conditions in Experiment 1 is indeed due to the differences in their logical status, and not on lower-level visual differences.

(iii) Exploring differences in logical representations in the Object Out phase of Experiment 1.

At Object Out, the permutation analyses showed that the conditions with logical valence (INF and LOI) elicited higher pupil dilation with respect to the visually matched conditions in which the identity of the objects was known (respectively, NOV and NON). Besides these planned contrasts, our scenes can also be used to explore the potential differences between conditions with different logical role: INF, when an inference can be drawn to determine the identity of the object in the cup, and LOI, where no inference can be drawn and the representation of an unknown object has to be maintained. However in this case the scenes are matched for temporal but not for visual features: in the former case the inference is triggered by the fact that the object behind the occluder exits fully visible, whereas in the latter condition the object exits the occluder remaining behind the grass, so as not to be identifiable (Fig. 1 A). The permutation analyses detected differences among INF and LOI in a time region posterior to that where the visual differences were located (Fig. 2 C), whereas in the visually equivalent, but logically irrelevant, NOV-NON contrast (Fig. 1 B) the differences were only present in that region (Fig. S2 A). This suggests that INF and LOI could elicit different representations or processes, and that inference making could come with an added with respect to simple retention of the representation of an unknown object (which, in itself, is different from that of a known object). In order to exclude any influence of the visual differences, however, a test of the difference of differences would be required. Thus, we ran a permutation analysis between the difference between INF and LOI, and the differences between NOV and NON (Fig. S4), with the same criteria as the permutations described above. No significant different was found. Thus, unlike what we found in the brain responses, the difference between INF and LOI at Object Out detected in oculomotor data cannot be uniquely attributed to the lo by differences in the logical nature of the two conditions.

Analysis of participants' choices in the Response phase

Additional analyses were performed on participants' choices at the Response phase. All participants but one (due to a material failure) were analyzed. These analyses were conducted to check whether participants were attentive to the scenes..

Participants' accuracy in the Response phase. The Mean accuracy in the Response phase, when participants had to indicate which object was inside the cup, was 86,8%. The percentage of missing responses was 3.9%. Accuracy per condition was 91% (SD=0.07) for INF, 78% (SD=0.15) for LOI, 93% (SD=0.09) for NOV, and 87% (SD=0.14) for NON. No participant scored below chance (33%). Non-parametric anova (Friedman test) showed differences between conditions (Fr=25.4, p=1.2e-5). Post-hoc comparison revealed that the effect was carried by the LOI condition, which differed from INF (p=0.0001), NOV (p=8.5e-5) and NON (p=0.027). No other differences were found. If we consider that the LOI condition is the only one that involves a disjunctive representation, or an existential quantifier to be maintained until the end of the scene, some extra errors are to be predicted.

2 Imaging data and analyses for Experiment 2

Procedure

In Experiment 2, participants undertook a short session of practice trials outside the acquisition room. We gave participants the exact instructions as in Experiment 1 to accomplish the task. Scenes were displayed in a 13-inch MacBookPro and after all scenes a response about the content of the cup was required. Only during this practice session was auditory feedback provided after each response. After eight consecutive accurate responses, the practice session ended and participants got ready for the imaging session.

During this session, stimuli were displayed on a MRI compatible LCD screen (BOLDSCREEN32 from Cambridge Research Ltd.). The sequence was controlled by a MacBookPro running PsyScope X build 88 (http://psy.cns.sissa.it/). Data were collected via PsyScope X, which also synchronized the movies to the internal timing of the scanner acquisition. Participant could record their responses via a 5-button non-magnetic response pad (Current Designs, Package 932 with Pyka HHSC-1×5-N4).

Magnetic Resonance imaging The acquisition was performed with a 3-Tesla whole body system (Siemens Trio), with a 32-channel head-coil. For each participant, the anatomical image was taken at the end of the session using a 3D gradient-echo sequence (MPRAGE, voxel size =1 x 1 x 1.1 mm, 160 slices. A total of 2200 functional scans were acquired during one single session of 440 scans per run (N=5), using an Echo-Planar sequence sensitized to the BOLD effect (TR=1.81 s,TE=30.4 ms, flip angle=71°, matrix= 64x64, voxel size=1.5 x 1.5 x 1.5 mm, 69 interleaved axial slices).

Data preprocessing and Analysis

Data processing was performed with SPM12 (Wellcome Department of Cognitive Neurology, software available at http://www.fil.ion.ucl.ac.uk/spm). Functional images were first realigned, corrected for slice timing differences and co-registered with the anatomy. The anatomy was segmented into grey and white matter and spatially normalized to the Montreal Neurological Institute (MNI) space using default parameters. The spatial normalization was applied to the functional images and smoothed with an isotropic Gaussian kernel (FWHM= 4 mm).

A general linear model was generated for each participant including 16 event-regressors obtained by crossing the four experimental conditions (INF, LOI, NOV, NON) and four events [Inter-stimulus interval (with duration ranging from ~0.8- 4.3s), Scooping phase (duration=0), Object Out phase (duration=0) and Motor response (duration=0)]. These sixteen variables were convolved by the canonical hemodynamic response function of SPM. Data were high-pass filtered with a cutoff of 160 s. Six motion regressors corresponding to translation and rotations along each axis were included in the design matrix. Group level analyses were performed using simple t-tests on individual contrast maps spatially smoothed with a Gaussian filter (*FWHM*=8 mm). All these analyses were performed using the Python module Nilearn (https://nilearn.github.io). FDR at 5% and uncorrected p <.001 voxel-wise methods were computed and the most conservative criterion for each contrast was retained.

Analysis of participants' choices in the Response phase

The mean accuracy and the proportion of missing responses were computed for all participants, except for two participants for whom, due to a technical failure, the button box failed to record.

Accuracy at identifying the object inside the cup. Average accuracy was 89%; 92.7% (SD=0.24) for INF, 85.3% (SD=0.28) for LOI, 94.7% (SD=0.17) for NOV and 85.6% (SD=0.28) for NON. The percentage of missing responses was 6%. No participant responded below chance. One-way non-parametric anova (Friedman test) showed statistical differences in accuracy between conditions ($Fr(3,19)=19.2$, $p=0.0002$. Post-hoc analyses revealed differences between NOV>INF ($p=0.036$), NOV>LOI ($p=0.031$), and NOV>NON ($p=0.01$), indicating that the easiest condition was the control NOV, where participants saw the identity of both objects in the two phases.

3 Regions of Interest and response profiles

We analyzed the response profiles in six Regions of Interest selected from previous brain imaging studies on verbal logical reasoning and language constituent structure. Regions in the frontal cortex (left Precentral [PrC] split into dorsal [BA6] and ventral [BA44]) and the Inferior Parietal lobe (IPL) were adopted from [5]; the posterior and anterior Inferior Frontal Gyrus (pIFG-BA44; aIFG-BA-47) from [6]; and the posterior superior temporal sulcus (pSTS), IFG orbitalis (IFGorb) and pars triangularis (IFGtri)] from [7]. All ROIs were provided by the authors. ROIs from Pallier et al. (2011) were spheres of 10mm radius centered on the first maxima of each cluster and intersected with the contrast of interest (increase in activation with constituent size). ROIs from [5] and [6] corresponded to the original areas found active in the contrasts of interest (sentences containing logical connectives vs baseline). After exploring the specific locations and surfaces of the ROIs, aIFG and pIFG selected from [6] where finally excluded from the analyses given the overlap with IFG dorsal (from [5]) and IFGorb (from [7]) respectively. The final selection appears in Fig.3.

To extract the time course of activations in Regions of Interest we also implemented a finite impulse response (FIR) model (with the onset at the beginning of the scenes) to better estimate the temporal course of activity with respect to what HRF can provide. This model was computed using SPM12 (https://www.fil.ion.ucl.ac.uk/spm/software/spm12/). Each condition was modeled using 16 time bins lasting 1.81 s each. Bins 6 and 7 correspond to the Scooping phase, bins 11 and 12 to the Object Out phase, and bins 13 and 14 to the preparation of the Response phase. They were average per participant, trial and condition before the analyses.

We first performed an ANOVA to assess the effect the condition within ROIs at both the Scooping and Object Out phases, and their interactions. Second, to asses lateralization in non-verbal reasoning we flipped the ROIs by using a Nilearn library (nilearn.image.swap_img_hemispheres(img)) and extracted the activation coefficients from the same brain images. Then we ran an ANOVA with lateralization, condition and phase as fixed main factors and participants as a random factor. All reported effects were Bonferroni-corrected for multiple comparisons. Table S9 displays the statistics computed with both models.

4 Exploratory analysis of the MPFC activity at the Scooping phase of Experiment 2

Whole brain analyses revealed activity in prefrontal areas (MPFC, BA10) at Scooping; we evaluated whether such an activity was specifically connected to logical representations. To do this, we extract the data from the FIR model defined as explained above and performed an exploratory repeated measures ANOVA with condition and phase as fixed main factors and participants as a random factor. We found an interaction effect between condition and phase ($F=21.4$, $p<0.0001$). Post-hoc comparisons showed that at the Scooping phase those conditions in which the object was unknown were more active than those in which it was known (both INF and LOI differed from NOV and NON at $p<0.0005$), while at Object Out the trend was the opposite, with the INF condition less active than both NOV and NON conditions (respectively, $p<0.03$ and $p<0.02$). These results indicate that the mPFC is active during the construction of a logical representation, but has no role during the application of a rule of inference (Table S9).

5 Exploratory analysis of the Default Mode and Multiple Demand Networks

The large fronto-parietal areas we reported as more active at Scooping in the Known condition, compared to the Unknown condition, is also compatible with a deactivation of the Default Mode Network (DMN) in the

Unknown condition. In it, participants have to wait for the Object Out phase in order to disambiguate the object in the cup, whereas they can actively identify it in the Known condition. Fronto-parietal activations are commonly associated with deactivations in the default mode network (DMN), which include the mPFC [8; 9]. A fronto-parietal activation also appeared at Object Out in the Inference condition, when participants can individuate the object inside the cup.

To evaluate the plausibility of this interpretation, we assessed the involvement of DMN by performing exploratory Regions-Of-Interest analyses in the areas of the network classified as the DMN in the Schaefer parcellation [10] (Fig. S5, *A;* N=90, all with size > 10 voxels). We extracted the response profiles from our FIR model at both Scooping and Object Out (respectively, bins 6 and 7 bins 10 and 11; and see § 3 for details) performing repeated measure two ways ANOVAs for each ROI. In particular, we looked for interactions between Condition and Phase as an indication of a potential involvement in our task with a profile predicted by the above interpretation. To increase the likelihood to identify potential activations in the DMN, we performed uncorrected analyses with a p<0.001 threshold. In 28 areas covering 34% of the voxel composing the DMN we found an interaction (Fig. S8, *B;* and Table S13). However, post hoc comparisons showed that only a few of the interpretable interactions were in the direction predicted by the interpretation, while most displayed the opposite pattern. We notice that also the pattern of activation we described in BA10, which is particularly relevant to our interpretation, was also opposite to the one predicted by such an account. Overall, these considerations suggest that DMN responded to our design only partially and in a conflicting way, as if its activation were orthogonal to the working of this network.

Also the involvement of the Multi Demand Network (MDN) may potentially offer an alternative account to the activation profile observed at Object Out. Such a network includes large portions of frontal and parietal areas that are recruited under "a diverse range of cognitively demanding tasks, including selective attention, working memory (WM), task switching, response inhibition, conflict monitoring, novel problem-solving, and many more" [9, p.4362]. A logical inference such as the one we studied can also be seen as a general-purpose operation triggering the task of object individuation when participants can infer its identity at the Object Out phase. Thus, we estimated the extent to which the network we reported for the deduction overlaps with or is subsumed by the MDN. For consistency with the analysis presented above (Fig.S8, A), we used the network reported in [11] as a mask into the Schaefer parcellation. We then extracted the response profiles from our FIR model in the two conditions of interest (INF and NOV) at Object out. A total of 179 areas composed the MDN network (Fig. S9, *A*); from them we excluded areas with size smaller than 10 voxels (N=57; Fig. S9, *B*). In the remaining 122 areas (Fig. S9), we computed the average of the activation time course and performed a repeated two ways ANOVA per ROI, with condition (INF and NOV) as fixed main factor and participants as a random factor, with the same criteria as above. Forty-four areas showed effects consistent with the results we reported; namely, they were more active in the INF than in the NOV condition (Fig. S9, *C*, and Table S14). This represents the 38% of the voxels composing the MDN. All other MND areas did not respond to the task. This suggests that, instead of being an overall activation of MDN induced by the task of object individuation, our conditions elicited a response for the particular logical operation required by it. Indeed, an elementary logical operation such as the one we focused on, being general and fundamental in many reasoning patterns, is likely to be involved in most of the tasks that elicit MDN activity. We thus see our results as a contribution to understand what the MDN actual does.

6 Exploratory analysis of the posterior BA10 / anterior BA47 reported in [1] for Experiment 2

We ran exploratory analyses centered in the anterior BA10/ posterior BA47 [-47 46 -2], an area reported in [1] as involved in verbal deductions. We first defined sphere of 10 mm of ratio and extracted the time course of activations estimated by our FIR model (see § 3). We then performed an exploratory repeated measures ANOVA with condition and phase as fixed main factors and participants as a random factor. We found no effect of Condition ($F(3,57)=0.02$, $p=0.99$) and no interaction effects ($F(3, 57)=1.5$, $p=0.22$). There was an effect of Phase ($F(3,57)=22.6$, $p=0.0002$) caused by the overall increase of activity from Scooping to Object Out in all the conditions. These results indicate that the BA47 area reported by Monti and collaborators has no role in our task.

References

1. Coetzee, J. P. & Monti, M. M. At the core of reasoning: Dissociating deductive and non-deductive load. Human Brain Mapping 39, 1850-1861 (2018).
2. Pesarin, F. Multivariate Permutation Tests with Applications in Biostatistics. Wiley, New York. (2001).
3. Guthrie, D. & Buchwald, J. S. Significance testing of difference potentials. Psychophysiology 28, 240-244 (1991).
4. Cesana-Arlotti, N. et al. Precursors of logical reasoning in preverbal human infants. Science 359, 1263 (2018).
5. Reverberi, C. et al. Neural basis of generation of conclusions in elementary deduction. Neuroimage 38, 752-762 (2007).
6. Baggio, G. et al. Multiple neural representations of elementary logical connectives. Neuroimage 135, 300-310 (2016).
7. Pallier, C., Devauchelle, A.-D. & Dehaene, S. Cortical representation of the constituent structure of sentences. PNAS Proceedings of the National Academy of Sciences of the United States of America 108, 2522-2527 (2011).
8. Hugdahl, K., Raichle, M. E., Mitra, A. & Specht, K. On the existence of a generalized non-specific task-dependent network. Frontiers in human neuroscience 9, 430 (2015).
9. Assem, M., Glasser, M. F., Van Essen, D. C. & Duncan, J. A domain-general cognitive core defined in multimodally parcellated human cortex. Cereb. Cortex 30, 4361-4380 (2020).
10. Schaefer, A. et al. Local-global parcellation of the human cerebral cortex from intrinsic functional connectivity MRI. Cereb. Cortex 28, 3095-3114 (2018).
11. Fedorenko, E., Duncan, J. & Kanwisher, N. Broad domain generality in focal regions of frontal and parietal cortex. Proceedings of the National Academy of Sciences 110, 16616-16621 (2013).

Supplementary Figures

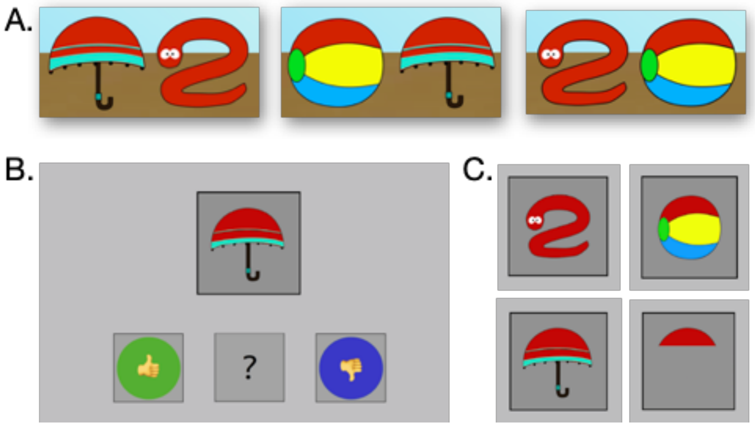

Fig. S1. Stimuli of Experiments 1 and 2. ***A***. Couples used in the scenes. Three single objects were paired to generate three couples (from left to right: umbrella-snake, ball-umbrella and snake-ball couples). Notice that all the objects share the same upper part such that when they are partially hidden their identity cannot be known. ***B.*** Screen during the Response phase. Participants had to indicate, with the proper device according to the experiment, whether the image depicted in the center of the screen (e.g., the umbrella) corresponded to the object inside the cup during the scene. From left to right: (i) Yes, it was; (ii) I cannot know; (iii) No, it was not. (C) Objects presented during the Response phase: snake, ball, umbrella and visible upper part. The visible upper part image was included to allow responses of the kind 'Yes, it was' during the Lack Of Inference trials (LOI) where the identity of the object in the cup cannot be ever known.

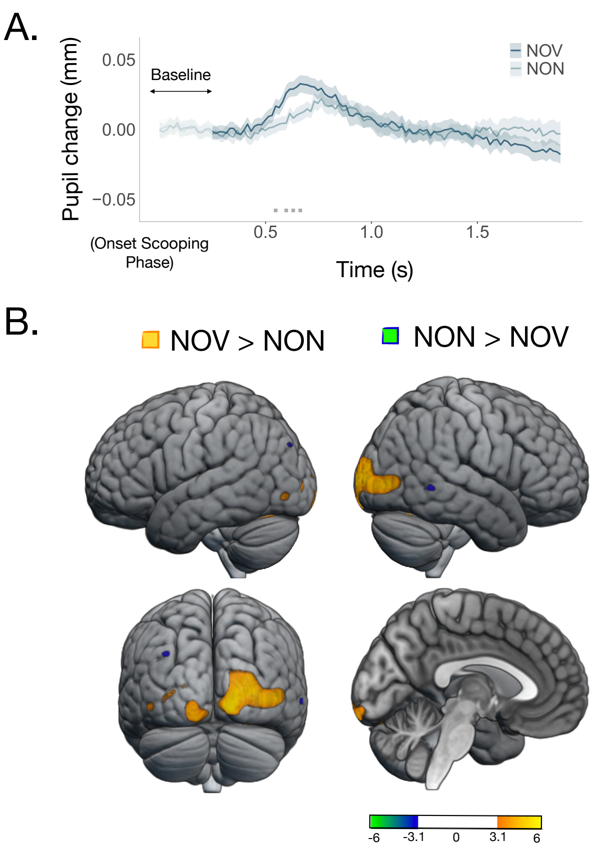

Fig. S2 Pupil dilation (Experiment 1) and Brain activations (Experiment 2) during the Object Out phase comparing the two No Inference conditions. At this stage, the No Inference conditions had same cognitive load but differed in the physical realization of the scene, either the object exited in front of the grass (NOV) or behind it (NON). ***A***. Temporal course of the mean pupil dilation from baseline in the NOV (indigo) and NON (blue stone) conditions. Participant's pupil did not differed between conditions (*p*=n.s), indicating that visual differences are not sufficient to generate a distinct pupil response in this phase. Shaded error areas: 95% within-participant CI. ***B***. Whole brain views showing activation for the NOV (orange/yellow) and NON (blue/green). Main differences in activation are located in visual areas (right Calcarine and left Lingual Gyrus), consistent with the differences in the visual inputs according to the condition (t=3.21, p <.001 uncorrected voxel-wise).

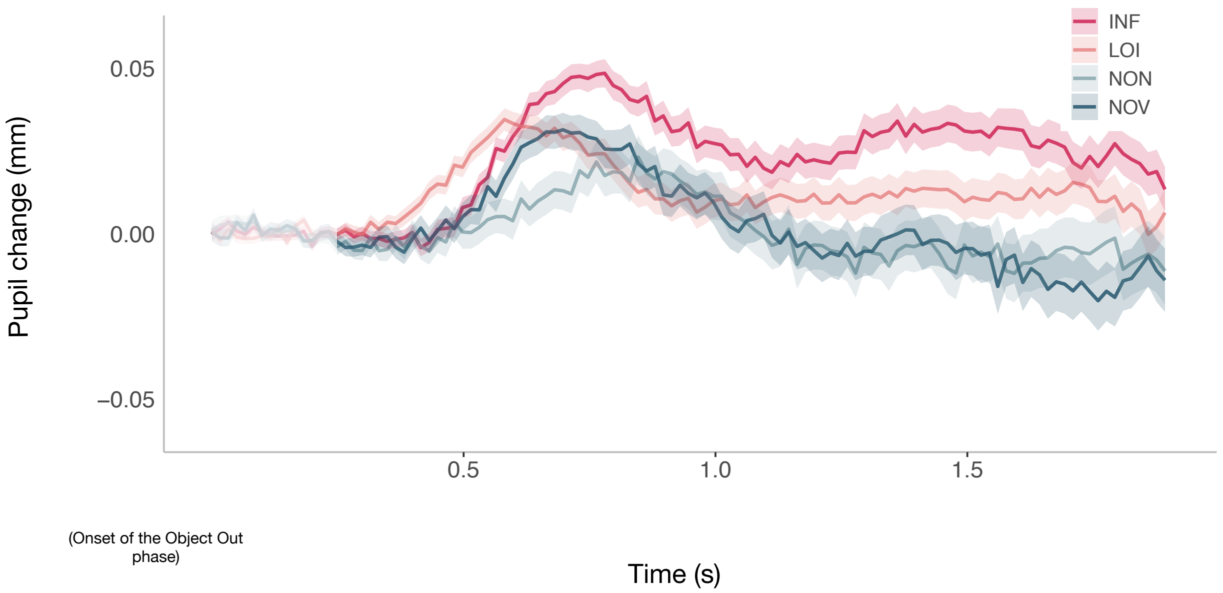

Fig. S3. Pupil dilation in all conditions of Experiment 1 during the Object Out phase. For easier comparison, Fig. S3 summarizes the analyzed oculomotor data at Object Out, when the object behind the occluder exits. Dependent on the condition, the exit may be informative to deduce the identity of the object inside the cup (INF- inference needed (red); LOI--no inference can be drawn (light red); NOV (blue) and NON(light blue)- no inference needed).Temporal course of the mean pupil dilation from baseline for all conditions. Shaded areas represent SE of the means.

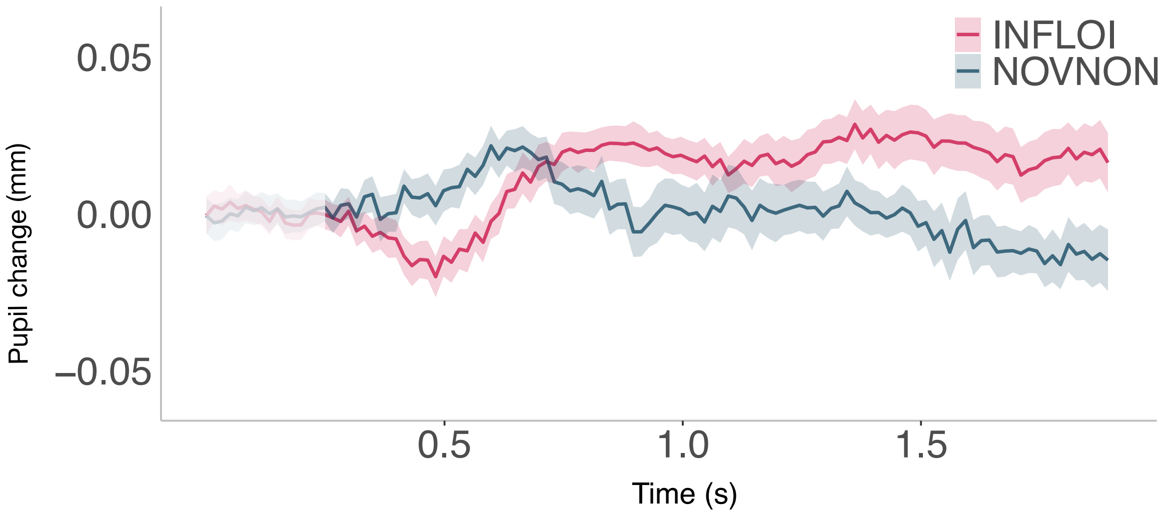

Fig. S4. Pupil dilation differences in the logical and nonlogical conditions of Experiment 1 during the Object Out phase. The graph presents the mean pupil dilation differences (SE) across the Object Out temporal window for the two conditions having logical valence (INFLOI: INF-LOI) and the two known conditions (NOVNON: NOV-NON). The permutation analysis revealed no time point in which the differences were significantly different.

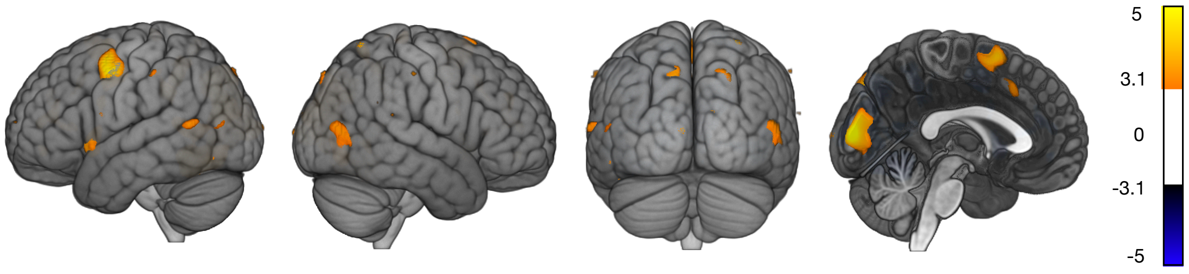

Fig. S5. Interaction (INF – LOI) – (NOV – NON) following the Object Out event.

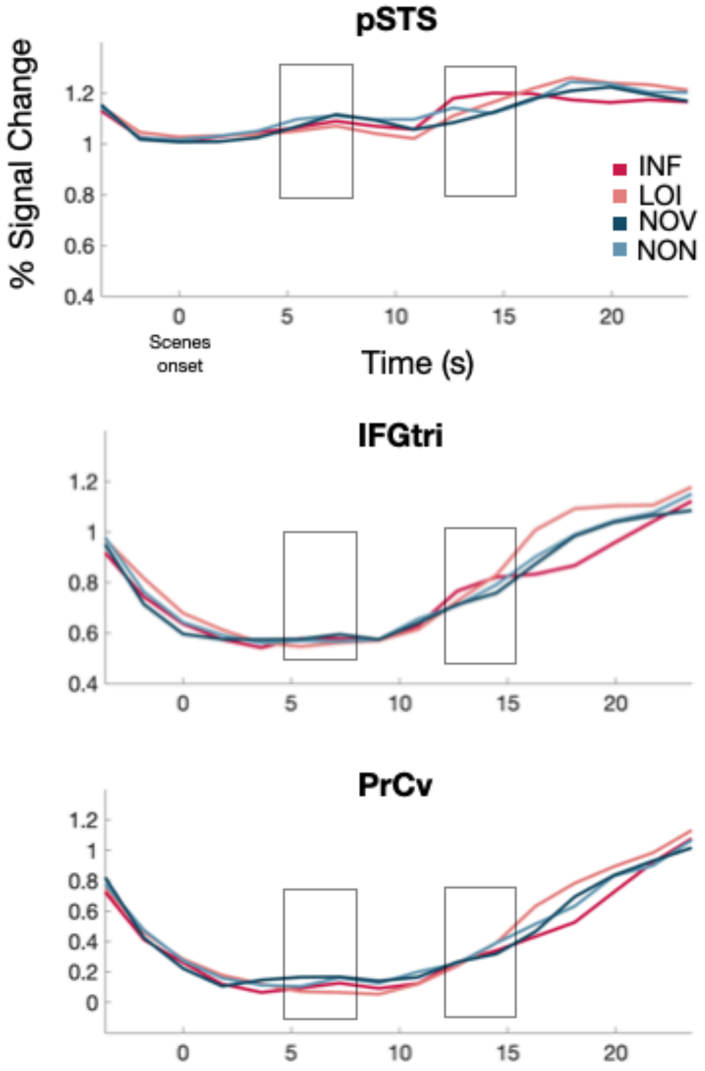

Fig. S6. Temporal course of brain activations in a priori Regions of interest (ROIs). Graphs depict mean coefficients in the posterior Superior Temporal Sulcus (pSTS), Inferior Frontal Gyrus triangularis (IFGtri) and Precentral ventral (PrCv) ROIs computed from the FIR model; x-axis extends from the trial onset to the Response phase included (total length: 28s). The Scooping phase extends from second 5 to 7.5, and the Object Out phase from 12 to 15.6 s (indicated by rectangular boxes). An ANOVA was performed to assess the effect of Phase (Scooping and Object Out) and Condition (INF, NOV, LOI and NON), and interactions within each ROIS (N=6) (see Table S7 for statistics). There were no interaction effects or main effects of Condition in these ROIs.

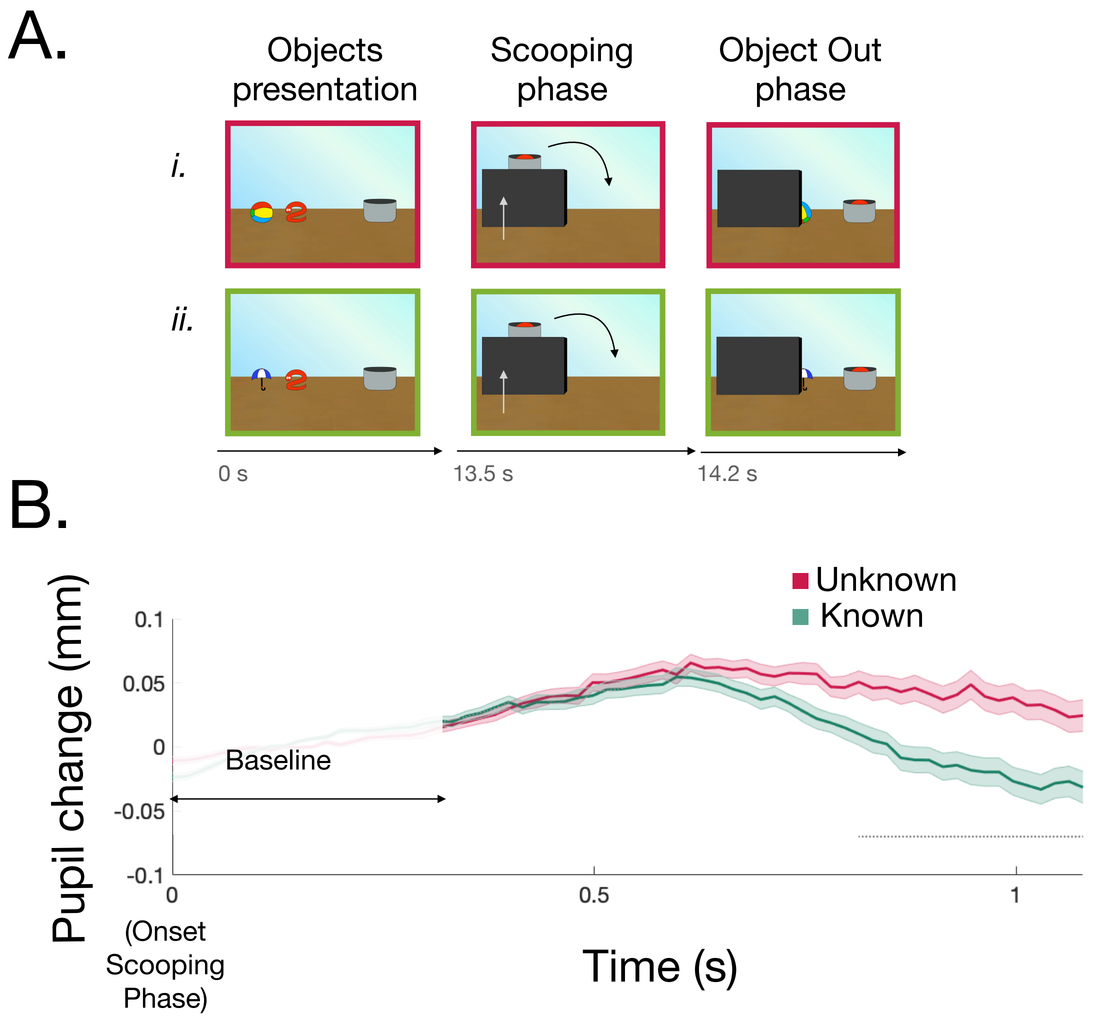

Fig. S7. Pupil temporal course during the Scooping phase in Cesana-Arlotti et al. (2018). **A**. Schematic presentation of the main events in scenes from Experiment 7. No Inference type 2 condition (ii) presented a couple of objects with different upper parts (e.g., blue umbrella & red snake). The realization of the scooping under this condition was identical to one of the Inference (i), but there was no ambiguity when the object was inside the cup. **B**. Mean pupil baseline-corrected during the Inference (unknown content inside the cup; red), and No Inference type 2 (known content; green) trials during the Scooping phase. Shaded areas: 95% confident interval. Permutation test revealed differences between conditions appearing from 813 ms to 1062 from the onset of the phase, with a higher pupil dilation when representing an unknown object inside the cup.

A.

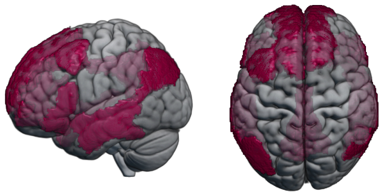

B.

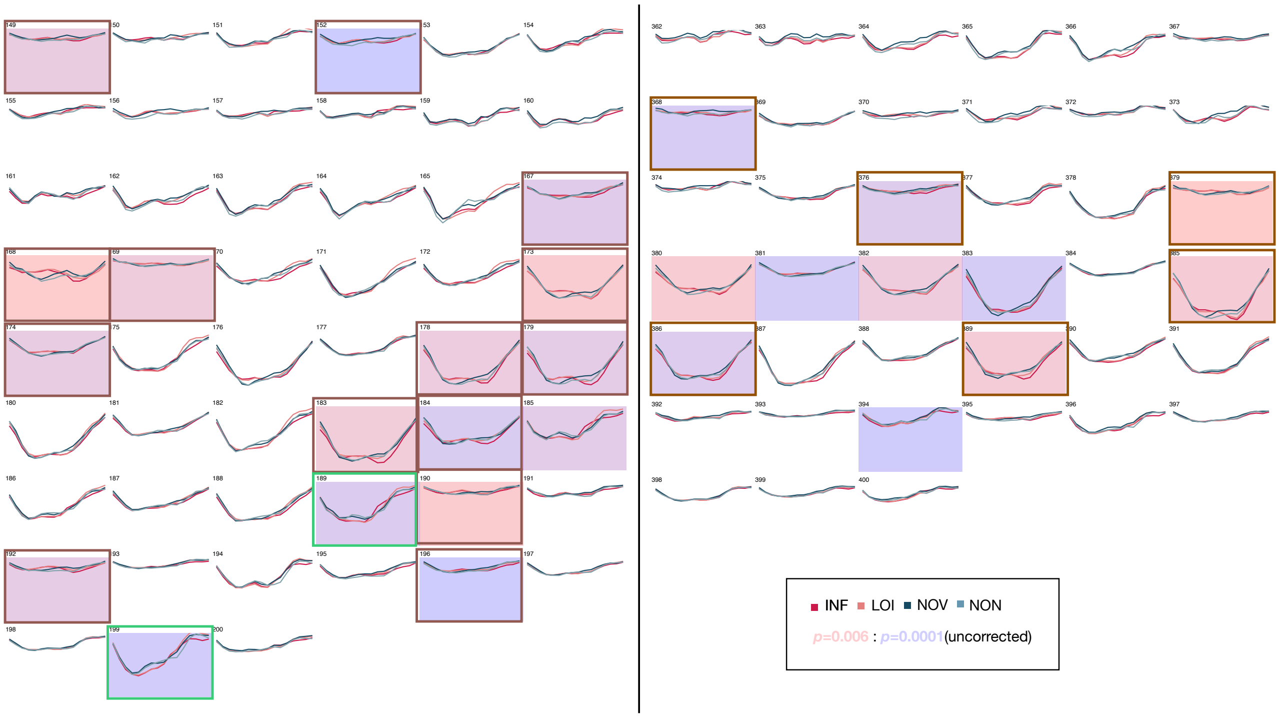

Fig S8. Default Mode Network ROIs analysis. (**A**) Left and superior views of the DMN from Schaefer parcellation in a rendered brain. (**B**) Plots of the temporal course of mean coefficients per ROI (N=90) computed from the FIR model (x-axis extends from the trial onset to the Response phase included (total length: 28s)). Plots on the left side of the black continuous line represent areas located in the left hemisphere. Plots on the right represent areas located in the right hemisphere. ANOVAs at each ROI were performed to asses the presence of interactions ($p<0.001$ uncorrected) between Phase (Scooping and Object Out) and Condition (INF, NOV, LOI and NON). Interactions were present in areas covering the 34% of the total voxels of the DMN. Shaded-color panels indicate the strength of the interactions (see table S11 for statistics). Colored rectangles code the pattern of the effects (Green rectangles indicates areas where the interaction depended on Unknown conditions (INF/LOI) being more active than Known conditions (NOV/NON) at Scooping but tending to invert activations at Object Out. Brown rectangles indicate the opposite pattern). Some of the interactions reported in the figure did not have significant post- hoc contrasts (N=8); in others post-hoc contrasts were significant, only in one of the Phases (at Scooping: N=1; at Object Out: N=16).

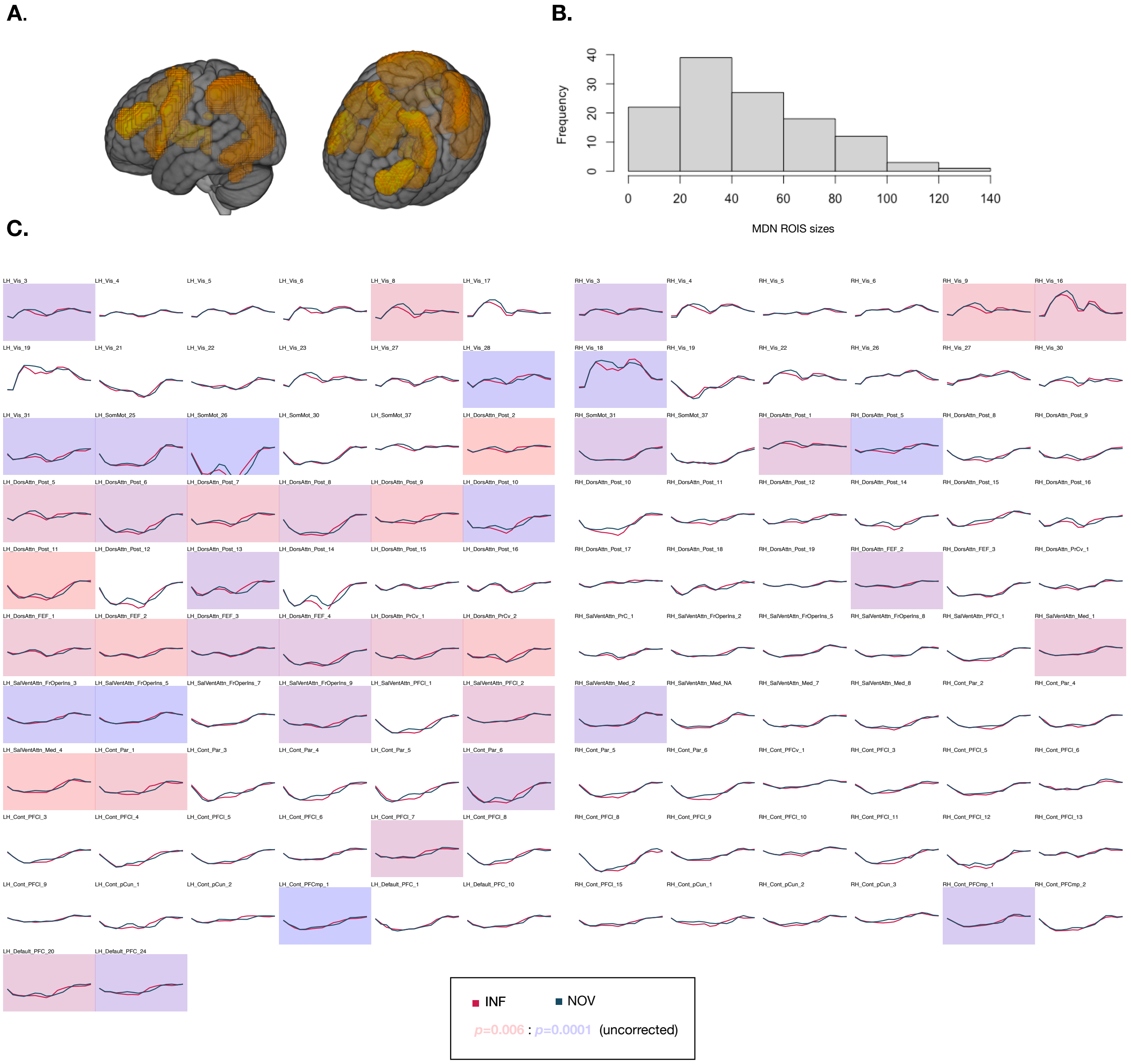

Fig S9. Multi Demand Network ROIs analysis. (**A**) Left and superior views of the MDN from Fedorenko et al. (2013) in a rendered brain. (**B**) Histogram for the ROI sizes after masking the MDN network onto the Schaefer parcellation (8). (**C**) Plots of the temporal course of mean coefficients (x-axis extends from the trial onset to the Response phase included (total length: 28s)) per ROI (N=122) computed from the FIR model. ANOVAs at each ROI were performed to asses the presence of differences between INF and NOV Conditions at Object Out. Difference ($p<0.001$ uncorrected) were found in areas covering the 38% of the MDN, with INF always more active than NOV. Shaded-color panels indicate the strength of the interactions (see Table S12 for statistics).

Supplementary Tables

Table S1. Regions showing effects in the Unknown > Known contrast at the Scooping phase. Anatomical labels were obtained with the Anatomical Automatic Labelling toolbox (http://www. cyceron.fr/web/ aal_anatomical_automatic_labeling.html).

Region	X	Y	Z	Peak Stat*	Cluster Size (mm3)
Lingual R (17)	9.0	-78.0	-3.0	5.292	6777
Lingual L (18)	-9.0	-78.0	-6.0	4.215	
Calcarine R (18)	12.0	-91.5	10.5	4.120	
Lingual R (17)	1.5	-67.5	7.5	3.763	
Frontal Sup Medial L (10)	-1.5	60.0	28.5	4.201	813
Temporal Pole Mid L (38)	-42.0	12.0	-33.0	4.151	212
Frontal Med Orb L (10)	-1.5	61.5	-3.0	3.863	1501
Cingulum Ant R (11)	1.5	30.0	-6.0	3.856	
Frontal Med Orb L (11)	0.0	45.0	-6.0	3.610	
Frontal Sup Medial L (10)	-9.0	63.0	15.0	3.674	135

*Significant peaks thresholded at t > 3.47, voxel-level FDR at p<0.05 corrected.

Table S2. Regions showing effects in the Known > Unknown contrast at the Scooping phase.

**Region**	**X**	**Y**	**Z**	**Peak Stat***	**Cluster Size (mm3)**
Occipital Inf L (18)	-24	-90	-6	6.238	35019
Occipital Inf L (19)	-40.5	-67.5	-6	6.144	
Occipital Inf L (19)	-45	-78	-4.5	6.122	
Lingual L (18)	-21	-94.5	-13.5	5.529	
Temporal Inf R (37)	45	-63	-6	5.926	48839
Fusiform R (37)	43.5	-51	-16.5	5.785	
Lingual R (18)	24	-91.5	-7.5	5.655	
Occipital Inf R (37)	43.5	-60	-13.5	5.539	
Parietal Inf L (40)	-27	-52.5	55.5	5.313	26503
Parietal Inf L (40)	-31.5	-42	48	4.955	
Parietal Inf L (40)	-34.5	-45	55.5	4.849	
Parietal Sup L (7)	-27	-61.5	55.5	4.805	
Precentral L (6)	-54	4.5	37.5	5.104	7445
Precentral L (6)	-46.5	1.5	54	4.611	
Supp Motor Area L (6)	-12	6	66	4.176	
Precentral R (44)	49.5	7.5	36	4.180	1103
Frontal Sup R (8)	21	10.5	58.5	4.101	1579
Frontal Inf Oper L (6)	-55.5	9	18	4.077	364
SupraMarginal L (40)	-60	-48	34.5	3.813	759

*Significant peaks thresholded at $t > 3.47$, voxel-level FDR at $p<0.05$ corrected.

Table S3. Regions showing effects in the Inference > No Inference- Object Visible contrast at the Object Out phase.

Region	X	Y	Z	Peak Stat*	Cluster Size (mm3)
Precentral L (6)	-37.5	-6	42	4.846	22504
Precentral L (6)	-46.5	-1.5	48	4.502	
Supp Motor Area L (6)	-3	0	66	4.170	
Frontal Sup L (6)	-25.5	-4.5	60	4.167	
Occipital Inf L (18)	-31.5	-87	-9	4.347	5774
Temporal Inf L (37)	-42	-55.5	-9	4.291	
Occipital Inf L (19)	-37.5	-78	-10.5	3.556	
Lingual L (18)	-19.5	-97.5	-16.5	3.246	
Parietal Sup L (7)	-22.5	-70.5	45	4.299	6243
Occipital Mid L (7)	-27	-79.5	27	4.100	
Precuneus L (7)	-19.5	-61.5	33	3.547	
Thalamus L	-3	-13.5	-1.5	4.164	3864
Thalamus L	-3	-25.5	-6	4.109	
Thalamus L	-7.5	-18	7.5	3.868	
Precuneus R (17)	24	-60	28.5	4.026	766
Parietal Inf L (40)	-36	-40.5	54	4.022	10138
Parietal Inf L	-36	-33	39	3.959	
Parietal Inf_L	-24	-36	42	3.844	
Parietal Inf L	-16.5	-34.5	49.5	3.820	
Temporal Inf R (37)	43.5	-54	-7.5	3.958	1154
Temporal Mid R (37)	46.5	-58.5	-1.5	3.796	
Lingual R (18)	22.5	-85.5	-4.5	3.865	1917
Occipital Inf R (19)	31.5	-82.5	-7.5	3.637	
Calcarine R (17)	24	-102	3	3.616	
Cerebelum_6_R	27	-57	-28.5	3.658	499
Temporal Inf R	15	-57	-33	3.459	165

Postcentral_R (3)	46.5	-24	45	3.443	337
Cingulum_Mid_R	6	7.5	33	3.428	178
Temporal_Mid_L (21)	-48	-46.5	10.5	3.382	256
Insula L	-25.5	24	9	3.375	671
Insula L	-31.5	33	13.5	3.363	
Insula L	-22.5	28.5	0	3.206	
Vermis 3	0	-45	-13.5	3.341	155
Supp Motor Area R (6)	10.5	7.5	66	3.303	131

*Significant peaks thresholded at $t > 3.09$, $p < .001$ uncorrected voxel-wise.

Table S4. Regions showing effects in the No Inference- Object Visible > Inference contrast at Object Out phase.

Region	X	Y	Z	Peak Stat*	Cluster Size (mm3)
Frontal Mid R (46)	48	52.5	0	4.367	2943
Frontal Inf Orb R (47)	48	40.5	-13.5	4.102	
Frontal Inf Orb R (47,38)	46.5	31.5	-18	3.784	
Frontal Sup Medial L (9)	1.5	48	46.5	4.230	5238
Frontal Sup R (9)	16.5	46.5	46.5	3.786	
Frontal Sup Medial R (8)	7.5	34.5	54	3.706	
Frontal Sup Medial L (8)	-6	36	51	3.687	
Angular R (39)	51	-63	46.5	4.137	1134
Frontal Inf Orb L (47)	-43.5	39	-10.5	4.123	1883
Frontal Inf Tri L (45)	-57	31.5	0	3.365	
Cerebelum Crus1 L	-42	-81	-33	3.910	239
Cerebelum Crus2 L	-34.5	-84	-33	3.824	
Frontal Mid R (46)	40.5	22.5	45	3.784	523
Temporal Pole Sup L (38)	-49.5	24	-16.5	3.630	135
Angular L (39)	-55.5	-63	36	3.456	273
Frontal Inf Orb R (38,47)	28.5	22.5	-22.5	3.454	158
Frontal Mid Orb R (47)	34.5	42	-10.5	3.419	151

*Significant peaks thresholded at $t > 3.09$, $p < .001$ uncorrected voxel-wise.

Table S5. Regions showing effects in the Inference > Lack of Inference contrast at the Object Out phase.

Region (INF vs LOI)	X	Y	Z	Peak Stat*	Cluster Size (mm3)
Occipital Mid L	-43.5	-75	-1.5	6.282	183576
Occipital Inf R	39	-82.5	-6	6.018	
Fusiform L	-36	-48	-18	5.839	
Precentral_L (6)	-54	-3	46.5	5.647	11289
Precentral_L	-28.5	-13.5	37.5	4.214	
Frontal_Mid_L	-43.5	6	58.5	4.141	
Cingulum_Mid_R	12	-24	42	5.374	18552
Parietal_Sup_L	-18	-52.5	48	4.576	
Parietal_Inf_L	-27	-49.5	49.5	4.426	
Cingulum Mid L	-15	-34.5	51	4.327	
Supp Motor Area L (6)	-3	4.5	63	4.982	16200
Cingulum Ant L	-4.5	21	31.5	4.806	
Cingulum Mid L	-12	9	33	4.260	
Cingulum Mid R	9	18	39	4.155	
Inferior Temporal R (BA20)	27	-22.5	-4.5	4.914	42315
Temporal Pole (L) (BA38)	-52.5	10.5	1.5	4.514	
Cingulate (25)	9	31.5	-4.5	4.088	708
Precentral_R (6)	51	-1.5	51	4.085	3537
Precentral_R	45	0	45	3.837	
Precentral_R	33	-9	45	3.831	
FusiformL	-34.5	-9	-31.5	3.652	175
Frontal Mid R	24	40.5	25.5	3.422	270
Occipital Sup L (7)	-13.5	-88.5	40.5	3.406	202
Cingulum Ant R	4.5	31.5	22.5	3.318	101
Pallidum L	-10.5	3	-6	3.276	67

*Significant peaks thresholded at $t > 3.09$, $p < .001$ uncorrected voxel-wise.

Table S6. Regions showing effects in the Lack of Inference > Inference contrast at the Object Out phase.

Region	X	Y	Z	Peak Stat*	Cluster Size (mm3)
Frontal Sup Medial L (9)	-4.5	51	48	3.675	340

*Significant peaks thresholded at t > 3.09, p <.001 uncorrected voxel-wise.

Table S7. Regions showing effects in the interaction (Inference >Lack of Inference) > (No Inference > Object Visible> No Inference - Object Not Visible) contrast at the Object Out phase.

Region	X	Y	Z	Peak Stat	Cluster Size (mm3)
Cuneus_R	8.0	-88.0	18.0	4.856	32496
Calcarine_L	4.0	-90.0	10.0	4.826	
Calcarine_L	-10.0	-84.0	12.0	4.318	
Occipital_Mid_L	-30.0	-76.0	24.0	4.192	
Precentral_L	-48.0	2.0	44.0	4.804	13016
Supp_Motor_Area_L	-4.0	8.0	62.0	3.969	
Frontal_Sup_L	-18.0	2.0	62.0	3.893	
Frontal_Mid_L	-32.0	2.0	56.0	3.634	
Insula_L	-44.0	12.0	-6.0	4.498	2368
Fusiform_L	-42.0	-62.0	-16.0	4.284	11912
Fusiform_L	-36.0	-72.0	-16.0	4.185	
Temporal_Inf_L	-46.0	-50.0	-10.0	4.063	
Fusiform_L	-44.0	-52.0	-20.0	4.015	
Precuneus_R	18.0	-48.0	50.0	4.148	1320
Parietal_Sup_R	28.0	-58.0	64.0	3.942	
Frontal_Mid_L	-22.0	38.0	18.0	4.109	1976
Frontal_Sup_L	-20.0	46.0	12.0	3.772	
Frontal_Inf_Tri_L	-30.0	32.0	10.0	3.340	
Insula_L	-26.0	24.0	8.0	3.337	
Parietal_Inf_L	-32.0	-46.0	42.0	3.969	3696
SupraMarginal_L	-42.0	-42.0	32.0	3.738	
Parietal_Inf_L	-62.0	-30.0	46.0	3.287	
Frontal_Sup_Orb_L	-24.0	46.0	-8.0	3.779	296
Cingulum_Mid_R	10.0	26.0	30.0	3.664	832
Frontal_Sup_Medial_L	-2.0	20.0	40.0	3.413	
Cingulum_Mid_R	8.0	18.0	40.0	3.253	

Region	X	Y	Z	Peak Stat	Cluster Size (mm3)
Frontal_Inf_Oper_L	-42.0	6.0	26.0	3.646	352
Lingual_R	18.0	-78.0	-2.0	3.616	4280
Fusiform_R	28.0	-76.0	-2.0	3.615	
Temporal_Mid_R	56.0	-68.0	6.0	3.378	
Temporal_Mid_R	52.0	-76.0	12.0	3.362	
Frontal_Mid_R	28.0	40.0	32.0	3.572	432
Temporal_Mid_R	38.0	-56.0	0.0	3.566	344
Precentral_R	18.0	-30.0	72.0	3.515	728

*Significant peaks thresholded at t > 3.01, p <.001 uncorrected voxel-wise.

Table S8. Regions showing effects in the interaction (No Inference > Object Visible> No Inference - Object Not Visible) > (Inference >Lack of Inference) contrast at the Object Out phase.

Region	X	Y	Z	Peak Stat*	Cluster Size (mm3)
Cerebelum_Crus2_L	-34	-76	-44	3.793	232

*Significant peaks thresholded at t > 3.01, p <.001 uncorrected voxel-wise.

Table S9. Regions-of-interest (ROI) post-hoc analysis.

	Interaction Phase x Condition				Condition				Interaction Phase x Condition x Lateralization			
	FIR		GLM		FIR		GLM		FIR		GLM	
ROIs	F	p corr	F	p corr	F	p corr	F	p corr	F	p corr	F	p corr
IFGorb	4.700	**0.038**	4.690	**0.010**	1.025	2.330	0.700	3.000	0.470	4.140	1.030	2.400
IFGTri	0.664	3.467	1.710	1.020	0.265	5.103	0.870	2.769	2.983	0.232	5.400	0.014
pSTS	1.995	0.750	0.600	3.600	1.623	1.160	3.700	0.099	2.275	0.533	3.290	0.160
PrC Dorsal	17.670	**0.001**	22.290	**0.001**	1.007	2.378	2.735	0.311	12.904	**0.001**	15.280	**0.001**
PrC Vental	9.570	5.400	12.670	1.800	0.308	4.915	2.090	0.667	1.010	2.367	1.210	1.800
IPS	0.130	**0.001**	1.400	**0.001**	0.880	2.400	4.582	**0.037**	5.546	**0.006**	6.100	**0.006**

All p-values are Bonferroni corrected for multiple comparison across the 6 ROIs. P-values highlighted in blue indicate trends ($p<0.05$ uncorrected).

Table S10. Post-hoc comparisons for the interaction between Condition and Phase found in the prefrontal areas (MPFC, BA10) obtained during the whole brain analyses at the Scooping phase.

Contrast	Difference	P-value
Scooping,LOI - Scooping,INF	0.0114	0.9929
Scooping,NON - Scooping,INF	-0.1708	**0.0006**
Scooping,NON - Scooping,LOI	-0.1822	**0.0002**
Scooping,NOV - Scooping,INF	-0.2294	**0.0000**
Scooping,NOV - Scooping,LOI	-0.2408	**0.0000**
Scooping,NOV - Scooping,NON	-0.0586	0.4997
ObjOut,LOI - ObjOut,INF	0.0497	0.6346
ObjOut,NON - ObjOut,INF	0.1258	**0.0172**
ObjOut,NON - ObjOut,LOI	0.0761	0.2689
ObjOut,NOV - ObjOut,INF	0.1138	0.0377
ObjOut,NOV - ObjOut,LOI	0.0641	0.4213
ObjOut,NOV - ObjOut,NON	-0.0120	0.9916

Table S11. Post-hoc comparisons for the triple interaction found in ROI PrC dorsal provided by Reverberi et al. (2007).

	Left hemisphere				Right hemisphere			
	Scooping Phase		Object Out Phase		Scooping Phase		Object Out Phase	
	Mean	P-value (corr)	Mean	P-value (corr)	Mean	P-value (corr)	Mean	P-value (corr)
LOI - INF	0.001	1.000	-0.091	**0.002**	0.014	0.923	-0.025	0.657
NON - INF	0.133	**0.000**	-0.155	**0.000**	0.068	**0.073**	-0.032	0.468
NON- LOI	0.132	**0.000**	-0.063	0.134	0.054	0.390	-0.007	0.990
NOV - INF	0.141	**0.000**	-0.230	**0.000**	0.105	**0.000**	-0.063	0.023
NOV - LOI	0.140	**0.000**	-0.138	**0.000**	0.092	**0.002**	-0.038	0.302
NOV - NON	0.009	0.978	-0.075	**0.026**	0.037	0.318	-0.031	0.470

P values are post-hoc Scheffe corrected and further corrected for multiple comparisons.

Table S12. Post-hoc comparisons for the triple interaction found in ROI IPL provided by Reverberi et al. (2007).

	Left hemisphere				Right hemisphere			
	Scooping Phase		Object Out Phase		Scooping Phase		Object Out Phase	
	Mean	P-value (corr)	Mean	P-value (corr)	Mean	P-value (corr)	Mean	P-value (corr)
LOI - INF	-0.014	0.981	-0.094	0.266	-0.001	1.000	0.056	0.388
NON - INF	0.198	**0.000**	-0.169	**0.000**	0.142	**0.004**	0.031	0.817
NON- LOI	0.212	**0.000**	-0.075	0.157497	0.143	**0.004**	-0.025	0.891
NOV - INF	0.218	**0.000**	-0.170	**0.000**	0.193	**0.000**	-0.020	0.945
NOV - LOI	0.232	**0.000**	-0.076	0.143187	0.193	**0.000**	-0.076	0.145
NOV - NON	0.020	0.942	-0.002	0.999964	0.051	0.483	-0.051	0.483

P values are post-hoc Scheffe corrected and further corrected for multiple comparisons.

Table S13. Regions of the Default Mode Network with interaction effects.

ROI	F- value	P- value (Uncorrected)
LH_Cont_Par_1	16.814	0.000609
LH_Cont_Par_6	11.107	0.003498
LH_Cont_PFCl_7	13.703	0.001514
LH_Cont_PFCmp_1	8.225	0.009848
LH_Default_PFC_20	14.156	0.001318
LH_Default_PFC_24	10.405	0.004450
LH_DorsAttn_FEF_1	16.185	0.000727
LH_DorsAttn_FEF_2	19.822	0.000273
LH_DorsAttn_FEF_3	11.912	0.002675
LH_DorsAttn_FEF_4	12.304	0.002353
LH_DorsAttn_Post_10	9.364	0.006444
LH_DorsAttn_Post_11	30.230	0.000026
LH_DorsAttn_Post_13	10.927	0.003719
LH_DorsAttn_Post_2	32.578	0.000017
LH_DorsAttn_Post_5	15.962	0.000774
LH_DorsAttn_Post_6	12.132	0.002489
LH_DorsAttn_Post_7	16.722	0.000625
LH_DorsAttn_Post_8	12.963	0.001906
LH_DorsAttn_Post_9	18.549	0.000380
LH_DorsAttn_PrCv_1	16.109	0.000743
LH_DorsAttn_PrCv_2	20.024	0.000260
LH_SalVentAttn_FrOperIns_3	9.161	0.006938
LH_SalVentAttn_FrOperIns_5	8.471	0.008968
LH_SalVentAttn_FrOperIns_9	11.336	0.003238
LH_SalVentAttn_Med_4	20.455	0.000233
LH_SalVentAttn_PFCl_2	15.121	0.000988
LH_SomMot_25	9.968	0.005188
LH_SomMot_26	8.471	0.008969
LH_Vis_28	8.689	0.008264
LH_Vis_3	10.495	0.004313
LH_Vis_31	9.536	0.006055
LH_Vis_8	16.430	0.000678
RH_Cont_PFCmp_1	10.059	0.005023

ROI	F- value	P- value (Uncorrected)
RH_DorsAttn_FEF_2	11.632	0.002934
RH_DorsAttn_Post_1	13.071	0.001843
RH_DorsAttn_Post_5	9.086	0.007132
RH_SalVentAttn_Med_1	15.297	0.000939
RH_SalVentAttn_Med_2	10.251	0.004697
RH_SomMot_31	11.188	0.003403
RH_Vis_16	16.383	0.000687
RH_Vis_18	9.243	0.006734
RH_Vis_3	10.747	0.003954
RH_Vis_9	17.152	0.000555

Table S14. Regions of the Multiple Demand Network with effects of Condition at Object Out

ROI ID	F- value	P value (Uncorrected)
149	7.844	0.000668
152	5.143	0.006499
167	8.521	0.000912
168	14.393	0.000025
169	9.709	0.000232
173	10.961	0.000049
174	7.612	0.000710
178	8.264	0.000570
179	8.323	0.000801
183	9.420	0.000112
184	5.974	0.002536
185	8.083	0.000802
189	8.100	0.001842
190	9.864	0.000035
192	6.651	0.000764
196	4.812	0.006483
199	5.925	0.004041
368	5.555	0.003012
376	7.300	0.000952
379	13.076	0.000014
380	11.347	0.000052
381	5.360	0.003720
382	7.885	0.000335
383	6.232	0.003279
385	10.516	0.000073
386	7.032	0.001205
389	9.979	0.000089
394	5.490	0.004147

Appendix 1. Participants' Instructions for Experiment 1

Below, we report the actual text participants read for Experiment 1. As the instructions were given in Spanish, we report the Spanish text.

En este experimento verás vídeos cortos. El experimento en sí tendrá una duración de unos 40 minutos y habrá tres bloques. Esto significa que después de ver 1/3 de los vídeos, habrá un breve descanso en el que podrás descansar un poco, luego verás otro tercio de los vídeos. Después de eso, seguirá el segundo descanso y, finalmente, verás el tercio restante de los vídeos.
En estos vídeos, verás un par de objetos de estos tres que realizan ciertas acciones. En algunos verás la serpiente y la pelota, la pelota y el paraguas o el paraguas y la serpiente, pero siempre solo dos de los tres. (mostrarlo con el mouse). Tu tarea será seguir lo que sucede en los vídeos. ¡Veamos cómo se ve un vídeo! Intenta prestar atención a los eventos que verás en el vídeo. (empezar vídeo)
Así que aquí puedes ver un paraguas y una serpiente. Hay también un oclusor y una copa. Ahora podemos ver la copa de nuevo. Y sale la serpiente. (Narrar lo que está sucediendo en los vídeos sin mencionar qué objeto está en la copa ni términos relacionados con lógica).
Entonces, así es como se ve un vídeo. Verás muchos de estos vídeos simples que van variar en estructura. Después de algunos de los vídeos, pero no después de todos, habrá una pregunta. No sabrás si viene una pregunta o no cuando veas el vídeo, así que trata siempre de prestar atención a las escenas para que puedas responder las preguntas lo mejor posible.
La pregunta siempre será la misma: ¿Qué hay dentro de la copa? Esto significa lo que había dentro de la copa en el momento en que se detuvo el vídeo. Siempre verás una imagen en el medio de la pantalla, por ejemplo aquí puedes ver la serpiente. Entonces, la pregunta ahora es: ¿Estaba la serpiente dentro de la copa? Podrás dar una respuesta pulsando las teclas correctas de este teclado que tendrás en la habitación contigo. Las teclas que mapearán el panel correspondiente presentado en la pantalla son las teclas HJK. La tecla H significa que sí, esto es correcto, el objeto que se muestra estaba dentro de la taza. La tecla J significa que no puedes saber si el objeto que se muestra estaba dentro de la copa o no. Pero ten cuidado, porque esto no es igual a "No recuerdo", "No puedo recordar" o "No presté atención". Esto significa que, según el vídeo que viste, es imposible saber qué objeto está dentro de la copa. La tecla K significa que no, este no es el objeto que estaba dentro de la copa. Intenta mantener los dedos sobre estos tres botones durante la tarea y responde lo más rápido posible, ya que hay un tiempo limitado, solo 2 s para dar una respuesta. Así que recuerda prestar siempre atención a la escena y responder rápido, pero con precisión. Si no estás 100% seguro de tu respuesta, es mejor responder algo que perderse la pregunta. Así que aquí, la pregunta es si la serpiente estaba dentro de la copa. ¿Recuerdas si esto es correcto o no? Aquí se puede ver un paraguas. ¿Estaba el paraguas dentro de la taza?
Y por supuesto, también es posible que durante las preguntas veas el tercer objeto, por ejemplo aquí la pelota, que ni siquiera estaba en la escena. Entonces, esta pregunta, por supuesto, sería incorrecta, pero es un recordatorio para ti de que siempre es importante prestar atención a las escenas.
Y a veces solo verás esta parte superior de un objeto. Esto significa que cualquiera de los tres objetos podría estar dentro de la copa, no importa cuál. La pregunta es solo si había un objeto en la taza cuando terminó el vídeo. Para todas las respuestas dadas, utiliza H, J, K.
¿Tienes alguna pregunta antes de continuar?
